# Evolutionary metabolic landscape from preneoplasia to invasive lung adenocarcinoma

**DOI:** 10.1038/s41467-021-26685-y

**Published:** 2021-11-10

**Authors:** Meng Nie, Ke Yao, Xinsheng Zhu, Na Chen, Nan Xiao, Yi Wang, Bo Peng, LiAng Yao, Peng Li, Peng Zhang, Zeping Hu

**Affiliations:** 1 School of Pharmaceutical Sciences, Tsinghua-Peking Center for Life Sciences, Beijing Frontier Research Center for Biological Structure, Tsinghua University, Beijing, 100084 China; 2 Department of Thoracic Surgery, Shanghai Pulmonary Hospital, Tongji University School of Medicine, Shanghai, 200433 China; 3 Institute of Metabolism and Integrative Biology, Fudan University, Shanghai, 200438 China; 4 Shanghai Qi Zhi Institute, Shanghai, 200030 China; 5 School of Life Sciences, Tsinghua University, Beijing, 100084 China

**Keywords:** Metabolomics, Cancer metabolism, Non-small-cell lung cancer

## Abstract

Metabolic reprogramming evolves during cancer initiation and progression. However, thorough understanding of metabolic evolution from preneoplasia to lung adenocarcinoma (LUAD) is still limited. Here, we perform large-scale targeted metabolomics on resected lesions and plasma obtained from invasive LUAD and its precursors, and decipher the metabolic trajectories from atypical adenomatous hyperplasia (AAH) to adenocarcinoma in situ (AIS), minimally invasive adenocarcinoma (MIA) and invasive adenocarcinoma (IAC), revealing that perturbed metabolic pathways emerge early in premalignant lesions. Furthermore, three panels of plasma metabolites are identified as non-invasive predictive biomarkers to distinguish IAC and its precursors with benign diseases. Strikingly, metabolomics clustering defines three metabolic subtypes of IAC patients with distinct clinical characteristics. We identify correlation between aberrant bile acid metabolism in subtype III with poor clinical features and demonstrate dysregulated bile acid metabolism promotes migration of LUAD, which could be exploited as potential targetable vulnerability and for stratifying patients. Collectively, the comprehensive landscape of the metabolic evolution along the development of LUAD will improve early detection and provide impactful therapeutic strategies.

## Abstract (web summary)

Metabolic reprogramming occurs during tumor progression. Here the authors decipher metabolic trajectories from preneoplasia to lung adenocarcinoma in tumor samples and identify plasma metabolites as potential predictive biomarkers for early detection.

## Introduction

Lung cancers remain the most common malignancy and the leading cause of cancer deaths worldwide^1^. Lung adenocarcinoma (LUAD) is the most common subtype^2^. Before progression to invasive LUAD, there is step-wise evolution of pre-neoplasia and pre-invasive lesions, ranging from atypical adenomatous hyperplasia (AAH) to atypical pre-invasive adenocarcinoma in situ (AIS), minimally invasive adenocarcinoma (MIA), and eventually invasive adenocarcinoma (IAC)^3^. Clinical application of low-dose computed tomography (CT) has contributed to a substantial increase in the detection of pulmonary nodules and a reduction in mortality of lung cancer, however, the high false-positive rate of imaging highlights the need for potent approaches for early detection of high-risk patients^4^. Recent studies have depicted the genomic and immune landscape of these lesions and revealed the molecular events underlying the initiation and progression of LUAD^5–10^, which may facilitate early diagnosis and cancer prevention. However, deep insight into the metabolic trajectory from AAH to IAC remains elusive.

Metabolic reprogramming has been regarded as a cancer hallmark and provides opportunities for cancer diagnostics, prognostics, and therapeutics^11,12^. In the past decade, metabolomics profiling has dramatically expanded our knowledge of metabolic alterations evolving during cancer development. For example, large-scale metabolomics analysis of clear cell renal cell carcinoma (ccRCC) reveals elevation of glutathione and dipeptides occurs during clinical progression and metabolomics clustering of human ccRCC identifies high-risk and low-risk subsets, which can afford complementary information for patient stratification^13^. Multi-omics data have showed that metabolic shifts occur from a very early stage of the colorectal cancer development^14^. Notably, sodium-dependent glucose transporter 2 (SGLT2) reportedly overexpressed in AAHs of the lung does not transport the common cancer imaging tracer ^18^fluoro-2-deoxyglucose (FDG) but the methyl-4-deoxy-4-[^18^F]-fluoro-α-D-glucopyranoside (Me4FDG) for identification of AAH and low-grade adenocarcinomas of the lung, suggesting a distinct metabolic signature in pre-neoplasia lesions^15^. Since much of what is currently known about the metabolic reprogramming of LUAD comes from the advanced stage, there is an urgent unmet need of characterizing metabolic evolution from pre-neoplasia to pre-invasive and invasive LUAD.

Here we perform targeted metabolomics profiling on both tissue and plasma samples derived from two independent cohorts to identify the metabolic alterations that may contribute to the initiation and progression of LUAD. We observe the progressive metabolic alterations from AAH to AIS, MIA, and IAC, suggesting that metabolic reprogramming emerges in premalignant lesions. Moreover, we reveal that different panels of circulating plasma metabolite biomarkers accurately distinguish early LUAD and benign diseases, which holds promise for non-invasive and low-cost early diagnosis. Notably, metabolomics clustering of IAC patients identifies three metabolic subtypes (S-I, S-II, and S-III) with distinct clinical characteristics. Pathway enrichment analysis shows that bile acid metabolism is aberrantly activated in S-III compared to S-I and S-II, and associates with poor prognosis. Functionally, we demonstrate that modulating bile acid metabolism can affect the migration of LUAD. Collectively, our findings decipher a comprehensive metabolic landscape from preneoplasia to invasive LUAD and provide an opportunity for more precise diagnostics and therapeutics of the disease.

## Results

### Metabolomic landscape of lung preneoplasia and adenocarcinoma

**Fig. 1:**
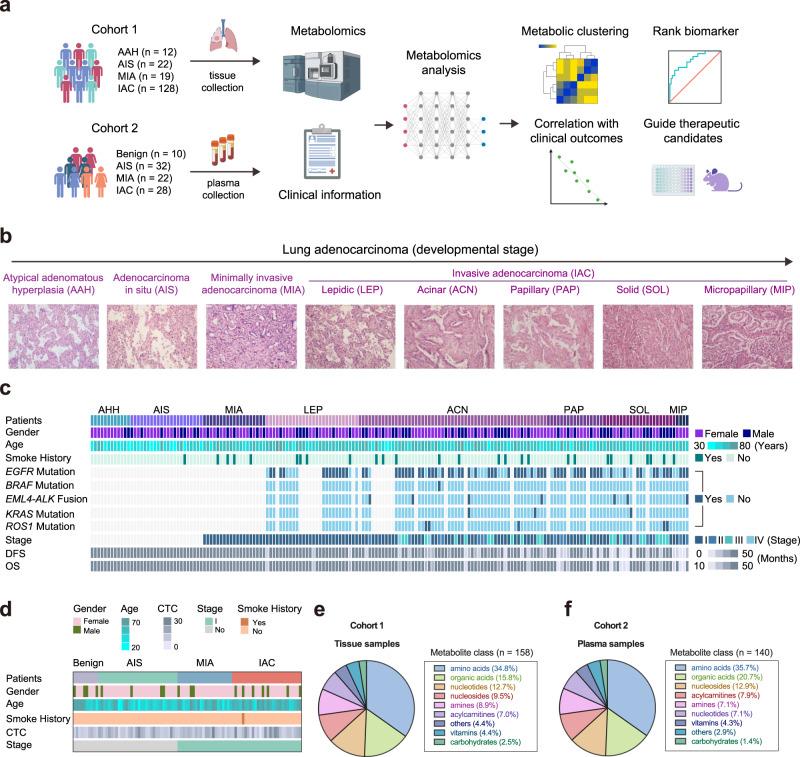
Schematic overview of the study. **a** Overview of the study design. The illustration was created with BioRender.com. **b** Eight morphological stages from lung preneoplasia to invasive adenocarcinoma. **c**, **d** Clinical parameters of the study cohort 1 (**c**) and cohort 2 (**d**) were indicated in the heatmap. DFS, disease-free survival; OS, overall survival; CTC, circulating tumor cell (FU/3 mL). **e**, **f** Classes and counts of metabolites detected in cohort 1 (tissue samples) and cohort 2 (plasma samples).

To obtain the comprehensive metabolomic landscape of the resected lesions from lung preneoplasia to pre-invasive and invasive adenocarcinoma, we performed the large-scale targeted liquid chromatography-mass spectrometry (LC-MS)-based metabolomics analysis on the tumor and paired normal adjacent tissue (NAT) obtained from a cohort (cohort 1) including 181 patients across different histological subtypes (*n* = 12 for AAH, *n* = 22 for AIS, *n* = 19 for MIA and *n* = 128 for IAC). In addition, we also characterized the dynamic alterations in circulating metabolites of plasma samples obtained from another independent cohort (cohort 2) including 92 patients across varying histological grades (*n* = 10 for benign diseases, *n* = 32 for AIS, *n* = 22 for MIA and *n* = 28 for IAC) (Fig. 1a). The clinical characteristics of the patients were shown in Fig. 1b–d and Supplementary Data 1. We totally detected 158 metabolites in the tissue samples and 140 metabolites in the plasma samples (Supplementary Data 2 and 3), covering a wide range of biochemicals including amino acids, organic acids, nucleotides, nucleosides, amines, acylcarnitines, vitamins, carbohydrates, and approximately 3–4% metabolites of other classes (Fig. 1e, f, and Supplementary Fig. 1a). Principal component analysis (PCA) showed the distinct metabolic profiles between tumor and NAT samples (Supplementary Fig. 1b). Of note, we identified 25 significantly differential metabolites in AAH versus its paired NAT, 67 significantly differential metabolites in AIS versus its paired NAT, 62 significantly differential metabolites in MIA versus its paired NAT, and 87 significantly differential metabolites in IAC versus its paired NAT (Wilcoxon signed-rank test, false discovery rate (FDR) < 0.05 and fold change > 1.25 or <0.8) (Supplementary Fig. 1c). The increasing number of differential metabolites in lesions of varying histological grades versus its NAT may suggest the progressively disturbed metabolic processes from pre-invasive to minimally invasive and invasive LUAD.

### The progressive metabolic evolution from AAH to IAC

**Fig. 2:**
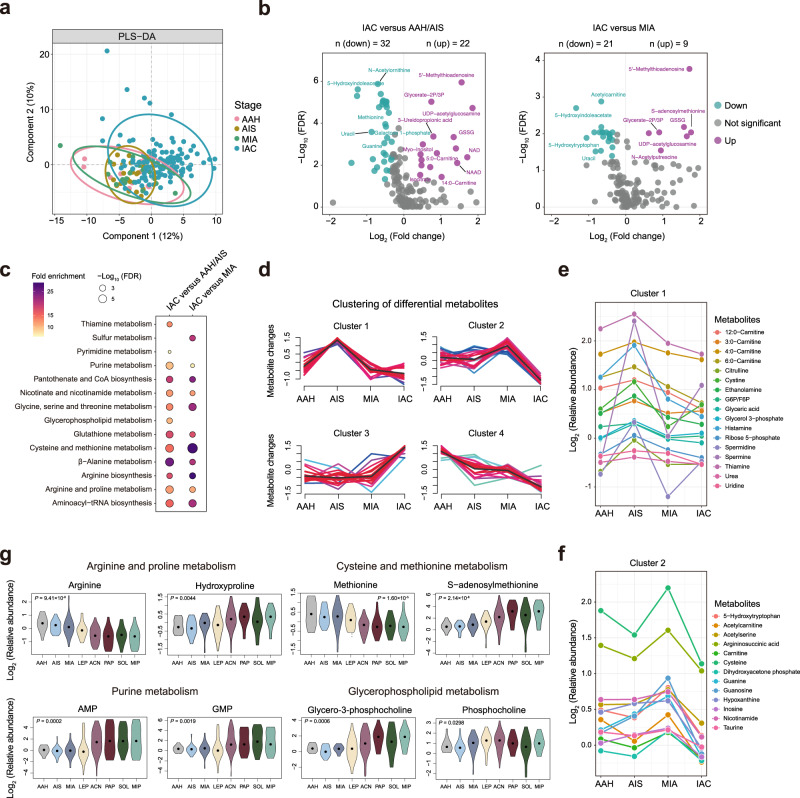
Progressive metabolic evolution from AAH to IAC. **a** Partial least squares discriminant analysis (PLS-DA) of the metabolomics data from AHH, AIS, MIA, and IAC patients. **b** Volcano plots of the significantly differential metabolites in pre-invasive group (AAH/AIS) versus IAC or in MIA versus IAC were shown. Two-sided Wilcoxon rank-sum tests followed by Benjamini-Hochberg (BH) multiple comparison test with false discovery rate (FDR) < 0.05 and fold change > 1.25 or <0.8. Metabolites significantly increased or decreased were colored in purple and green, respectively. **c** Kyoto Encyclopedia of Genes and Genomes (KEGG) metabolic pathways enriched by significantly differential metabolites in pre-invasive group (AAH/AIS) versus IAC or in MIA versus IAC group. One-sided Fisher’s exact test followed by BH multiple comparison test with FDR < 0.05. **d** Clustering of metabolic trajectories using differential metabolites among AAH, AIS, MIA, and IAC. Two-sided Kruskal-Wallis tests followed by BH multiple comparison test with FDR < 0.1. **e**, **f** Dynamic alterations of metabolites in cluster 1 and cluster 2. The dots represent the mean log_2_ relative abundance. Two-sided Kruskal-Wallis tests followed by BH multiple comparison test with FDR < 0.1. **g** Violin plots of metabolites in cluster 3 and cluster 4. The difference of metabolites among AHH, AIS, MIA, and IAC were evaluated using a two-sided Kruskal-Wallis test. Black dots represent population medians.

Given that metabolic reprogramming is crucial for cancer initiation and progression^12^, we were thus interested to delineate the progressive metabolic evolution from preneoplasia to AIS, MIA, and then IAC. Partial least squares discrimination analysis (PLS-DA) clearly distinguished invasive LUAD from pre-invasive and minimally invasive LUAD (Fig. 2a). Interestingly, we found 54 significantly differential metabolites in IAC versus AAH/AIS patients whereas 30 differential metabolites in IAC versus MIA patients (Wilcoxon rank-sum test, FDR < 0.05 and fold change > 1.25 or <0.8) (Fig. 2b), implying that metabolic processes may be gradually shaped during the early carcinogenesis of LUAD. Furthermore, Kyoto Encyclopedia of Genes and Genomes (KEGG) pathway-based analysis showed several metabolic pathways, such as nicotinate and nicotinamide metabolism, β-Alanine metabolism, glutathione metabolism, arginine and proline metabolism, were progressively disturbed in MIA and IAC, compared to pre-invasive lesions (Fig. 2c). In addition, purine and pyrimidine metabolism, thiamine metabolism, and glycerophospholipid metabolism showed vigorous perturbation early in the MIA before the invasive LUAD (Fig. 2c). To delineate the metabolite trajectories in the airway progress from preneoplasia to invasive LUAD, we performed c-means clustering using the differential metabolites among four stages (Kruskal-Wallis tests, FDR < 0.1) and identified four distinct clusters (Supplementary Fig. 2a and Fig. 2d). Metabolites in cluster 1 showed a marked increase in AIS (e.g., spermine, spermidine, histamine, and cystine) whereas metabolites in cluster 2 were specifically enriched in MIA (e.g., guanosine, guanine, cysteine, nicotinamide, and taurine) (Fig. 2e, f), suggesting that unique metabolic vulnerabilities may appear in specific early stages which could be exploited to diagnose and monitor premalignant lesions. Notably, several metabolites in cluster 3 and cluster 4 showed a stepwise increase or decrease from pre-invasive to MIA and IAC patients. The level of arginine was decreased whereas the levels of two key metabolites in arginine and proline metabolism, hydroxyproline and N-Acetylputrescine, were both moderately increased (Fig. 2g and Supplementary Fig. 2b). We also observed that S-adenosylmethionine (SAM) and 5’-methylthioadenosine that involved in cysteine and methionine metabolism displayed a constant upward trend, whereas methionine exhibited a downward trend along with the tumor progression (Fig. 2g and Supplementary Fig. 2b). These changes in metabolites involved in cysteine and methionine metabolism were consistent with the previous reports that perturbation of methionine metabolism plays a critical role in epigenetic modifications, such as methylation of DNA and histones, that promote tumorigenesis^16–18^. Moreover, a marked elevation in AMP and GMP, key metabolites associated with purine metabolism, were found in the IAC group compared to the pre-invasive and MIA patients, indicating that purine metabolism may be highly involved in the invasiveness during tumor development (Fig. 2g). In addition, we also found glycerol-3-phosphocholine and phosphocholine involved in glycerophospholipid metabolism increased progressively along with disease progression (Fig. 2g). Taken together, these findings depict the metabolic trajectory from AAH to IAC, and indicate that several reported metabolic vulnerabilities in invasive LUAD have emerged in premalignant lesions.

### Diagnostic value of circulating plasma metabolites in patients with different stages

**Fig. 3:**
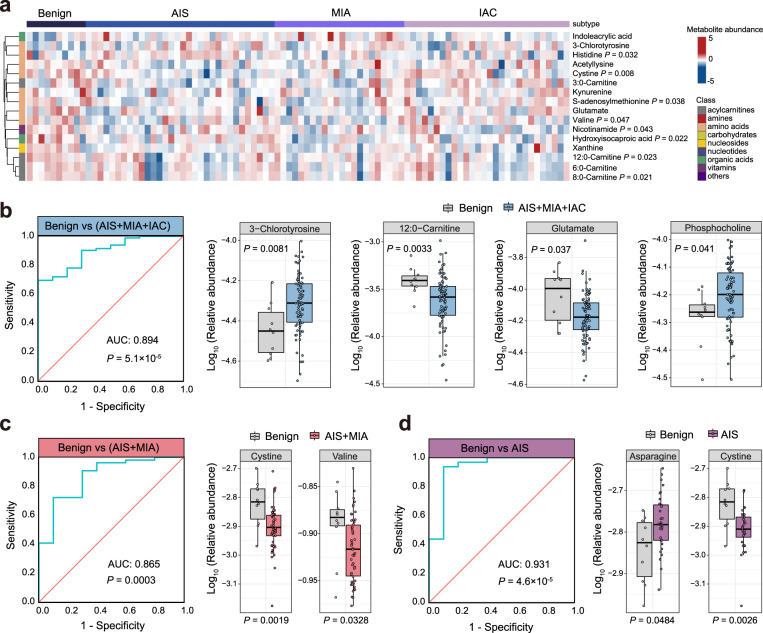
The alteration of circulating metabolites in plasma from patients with different stages. **a** Heatmap of metabolite alterations from benign diseases, AIS, MIA, and IAC. Two-sided Kruskal-Wallis tests with *P* < 0.1. **b** The receiver operating characteristic (ROC) curve and log_10_ relative abundance of 3-chlorotyrosine, 12:0-carnitine, glutamate, and phosphocholine (benign diseases, *n* = 10; AIS/MIA/IAC, *n* = 82). A two-sided Wilcoxon rank-sum test was used. **c** The ROC curve and log_10_ relative abundance of cystine and valine (benign diseases, *n* = 10; AIS/MIA, *n* = 54). A two-sided Wilcoxon rank-sum test was used. **d** The ROC curve and log_10_ relative abundance of asparagine and cystine (benign diseases, *n* = 10; AIS, *n* = 32). A two-sided Wilcoxon rank-sum test was used. In the box plots b–d, the center line represents the median, and the box bounds represents the inter-quartile range. The whiskers span 1.5-fold the inter-quartile range. AUC, Area Under Curve.

Early detection greatly enhances the chances for successful cancer treatment^19,20^. In contrast to the traditional biopsy and radiologic screening by low-dose CT, a blood-based test is non-invasive and relatively low-cost^21^. Identifying the circulating metabolic biomarkers is a promising approach that could facilitate blood-based screening for early LUAD. Therefore, we performed the plasma metabolomics profiling and totally identified 16 altered metabolites among four stages (Kruskal–Wallis tests, *P* < 0.1) (Fig. 3a). To discover predictive biomarkers for distinguishing different histological subtypes of LUAD, especially for early stages, we built logistic regression models based on differential metabolites between two given groups (benign diseases versus AIS/MIA/IAC, benign diseases versus AIS/MIA, benign diseases versus AIS). A biomarker panel of four metabolites with Area Under Curve (AUC) of 0.894, enabling the discrimination between benign diseases and LUAD groups (AIS/MIA/IAC), was identified (Fig. 3b). The panel consisted of 3-chlorotyrosine, 12:0-carnitine, glutamate, and phosphocholine. The levels of 3-chlorotyrosine and phosphocholine were significantly increased whereas the levels of glutamate and 12:0-carnitine were markedly decreased in LUAD groups compared to benign diseases (Fig. 3b). Interestingly, the marked decrease in glutamate in AIS/MIA/IAC compared to the benign diseases is consistent with a previous finding that plasma concentration of glutamate allows discrimination between lung cancer and lung inflammation, and a low glutamate concentration is considered as a diagnostic biomarker for lung cancer^22^. Moreover, in the classification of benign diseases and AIS/MIA, a combination of two metabolites showed a high AUC value of 0.865. The levels of two metabolites, including cystine and valine, were decreased in AIS/MIA compared to benign diseases (Fig. 3c). Notably, a panel of two metabolites (i.e., asparagine and cystine) achieved an AUC of 0.931 in distinguishing AIS from benign diseases. An increase in asparagine and a decrease in cystine were observed in AIS compared to benign diseases (Fig. 3d). These findings demonstrate the power of metabolomics for biomarker discovery, which facilitates early detection of LUAD more precise and accessible.

### Metabolic stratification of IAC patients and their clinicopathologic correlations

**Fig. 4:**
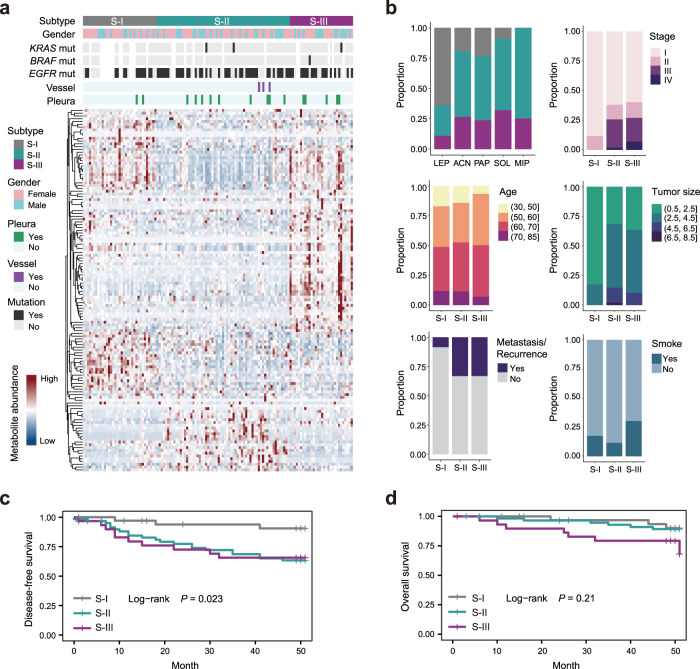
Metabolic stratification of IAC patients and their clinicopathologic correlations. **a** Heatmap indicating the relative abundance of metabolites in the identified three metabolomics subtypes. **b** Clinical parameters of each metabolic subtype were presented. Fisher’s exact test was used. **c**, **d** Association of three metabolic subtypes with clinical outcomes including disease-free survival and overall survival in IAC patients. A two-sided log-rank test was used.

To evaluate whether the invasive LUAD could be partitioned into subtypes with distinct metabolic properties, we performed unsupervised clustering analysis on IAC patients (Supplementary Fig. 3a, b). Of note, three metabolic subtypes with distinct clinical features were identified. We found that S-II had the highest mutation frequency of EGFR whereas S-I had the lowest (Fisher’s exact test, *P* = 0.023). Patients with KRAS mutation were observed in S-II and S-III, and patients with BRAF mutation were observed only in S-III (Fig. 4a). Intriguingly, the early-stage (*P* = 0.006) and lepidic pathological subtypes (*P* = 0.001) were enriched in patients in S-I, whereas solid and micropapillary pathological subtypes as well as higher level of tumor node metastasis (TNM) stages were enriched in patients in S-II and S-III. Moreover, clinicopathologic factors such as smaller tumor size (<2.5 cm, *P* = 2.39 × 10^−5^) and non-metastatic (*P* = 0.016) patients were more prominent in S-I versus the other two subtypes. Among these three metabolic subtypes, S-III had the highest percentage of smokers (*P* = 0.088) (Fig. 4b). Together, these data suggest that patients in S-II and S-III represent more malignant characteristics than those in S-I. To further assess the prognostic value of the metabolomics clustering, we examined the disease-free survival (DFS) and overall survival (OS) of patients in three metabolic subtypes. Consistent with the above observations, patients in S-I had the best prognosis whereas patients in S-II and S-III had the relatively poor prognosis (log-rank test, *P* = 0.023 for DFS) (Fig. 4c, d), indicating that the metabolomics clustering may precisely stratify the patients with different clinical outcomes.

Recent studies have established the comprehensive landscape of LUAD at genomics, transcriptomics, proteomics, and phosphoproteomics levels^23–25^, which prompted us to integrate our metabolomics data with relevant proteomics and transcriptomics data of invasive LUAD, thus facilitating the identification of the dysregulated metabolic pathways in different layers. Specifically, we jointly analyzed The Cancer Genome Atlas (TCGA) LUAD dataset, and transcriptomics as well as proteomics data downloaded from the published study^23^. Interestingly, we identified the common metabolic perturbations in tumor versus NAT samples based on multi-layer omics data. KEGG pathway enrichment analysis based on metabolomics data and published transcriptomics as well as proteomics data consistently showed that the glutathione metabolism was markedly disturbed (Supplementary Fig. 4a–c). Moreover, pathway analysis based on metabolomics data and published transcriptomics data as well as TCGA LUAD dataset consistently revealed a significant dysregulation of arginine-related pathways, including arginine biosynthesis and arginine and proline metabolism (Supplementary Fig. 4a, c, d). Therefore, we constructed a metabolic map detailing the alterations in mRNA, protein, and metabolite levels and observed that several metabolite changes were consistent with their putative metabolizing enzyme (Supplementary Fig. 4e), demonstrating that our metabolomics data were integrated well with other omics data which provided the cross-validated metabolic reprogramming.

Given that mutation of oncogene or tumor suppressor is a key driver in cancer cell-autonomous metabolic reprogramming, we therefore sought to determine the effect of EGFR mutation on the metabolomics profile of IAC patients. PCA analysis showed comparable metabolomic profiles between EGFR-mutant and EGFR-wild type (WT) tumors (Supplementary Fig. 5a), however, we found a small amount of metabolites altered in EGFR-mutant versus EGFR-WT tumors with *P* < 0.05, which did not pass the FDR cutoff. Cysteine showed the increased abundance whereas succinate, XMP, ribose 5-phosphate, glycerol 3-phosphate, glucarate showed the decreased abundance in EGFR-mutant tumors compared with EGFR-WT tumors (Supplementary Fig. 5b).

**Perturbation of bile acid metabolism is associated with poor prognosis, and promotes migration of LUAD**

**Fig. 5:**
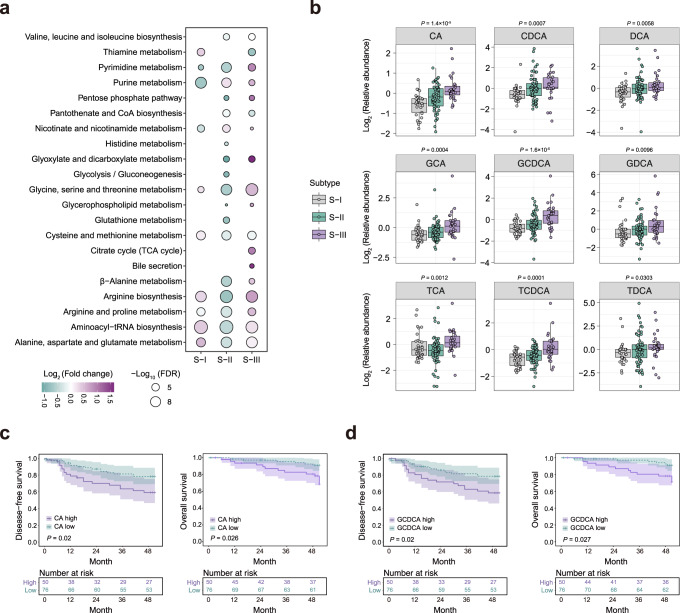
Accumulation of bile acids in metabolic subtype III and its association of with clinical outcome. **a** KEGG metabolic pathways enriched by significantly differential metabolites (Two-sided Wilcoxon rank-sum test, FDR < 0.05) in each subtype relative to the other two metabolic subtypes. One-sided Fisher’s exact test followed by Benjamini-Hochberg (BH) multiple comparison test with FDR < 0.05. **b** Boxplots of the log_2_ relative abundance of bile acids in three metabolic subtypes (S-I, *n* = 33; S-II, *n* = 63; S-III, *n* = 30). Two-sided Kruskal-Wallis tests were used. The centerline represents the median, and the box bounds represent the inter-quartile range. The whiskers span 1.5-fold the inter-quartile range. **c**, **d** Kaplan–Meier curves predicting the disease-free survival and overall survival of IAC patients stratified by bile acid level with two-sided log-rank *P* value. The patients were divided into high and low groups by 0.4 quantile of the bile acids levels in IAC patients.

After stratifying patients according to metabolic subtypes, we sought to determine the metabolic vulnerabilities of each distinct subtype. KEGG pathway enrichment analysis based on differential metabolites in each cluster showed that S-I with a good prognosis presented less metabolic perturbations compared with S-II and S-III with poor prognosis (Fig. 5a). Notably, S-III was specifically characterized by the highest levels of metabolites in pathways related to bile acid metabolism and citrate cycle (Fig. 5a). To further investigate the alteration of bile acids in the three distinct subtypes, we detected different bile acid species and found a marked increase in cholic acid (CA), taurochenodeoxycholic acid (TCDCA), glycochenodeoxycholic acid (GCDCA), and a moderate increase in deoxycholic acid (DCA), glycocholic acid (GCA), chenodeoxycholic acid (CDCA), glycodeoxycholic acid (GDCA), taurocholic acid (TCA) and taurodeoxycholic acid (TDCA) from S-I to S-III (Fig. 5b). Importantly, we identified that several bile acids, including CA, GCDCA, TCDCA, and GCA, were highly correlated with clinical outcomes. Patients with higher levels of bile acids showed poorer DFS or OS (Fig. 5c, d, and Supplementary Fig. 6a–e).

**Fig. 6:**
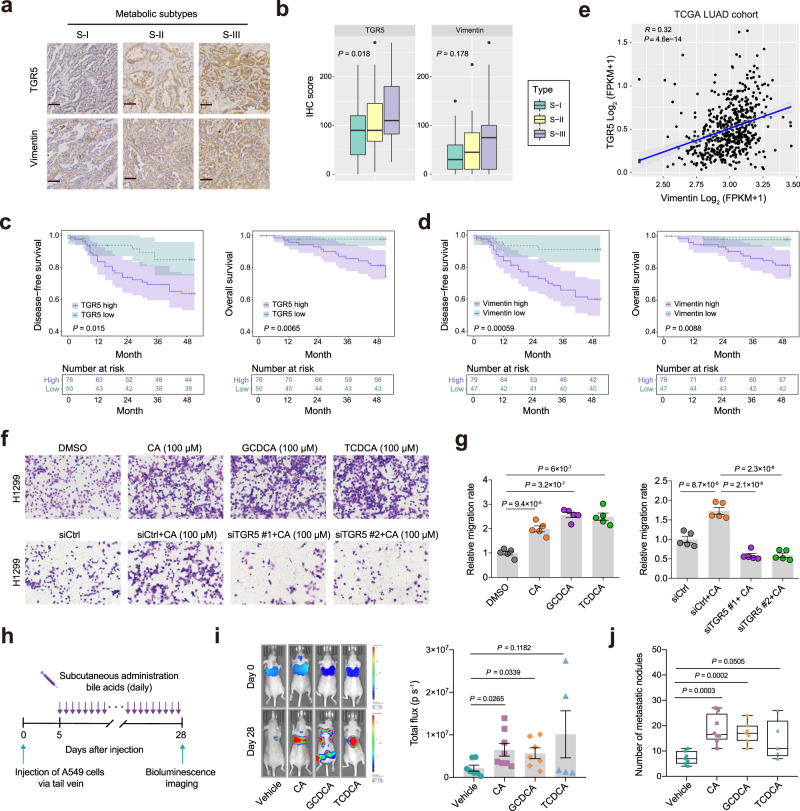
Aberrant bile acid metabolism promotes migration of invasive LUAD. **a** Representative image of immunohistochemical staining of TGR5 and vimentin on 126 IAC patient tumor from S-I (*n* = 33), S-II (*n* = 63), and S-III (*n* = 30) subtypes. Scale bar, 100 μM. **b** Immunohistochemical (IHC) score of TGR5 and vimentin from patients in S-I (*n* = 33), S-II (*n* = 63), and S-III (*n* = 30) subtypes. A two-sided One-way ANOVA test was used. The centerline represents the median, and the box bounds represent the inter-quartile range. The whiskers span 1.5-fold the inter-quartile range. **c** Kaplan-Meier curves comparing the disease-free survival and overall survival in IAC patients with a high group (histoscore > 80 in TGR5 expression) versus low group (histoscore ≤ 80 in TGR5 expression). A two-sided log-rank test was used. **d** Kaplan-Meier curves comparing the disease-free survival or overall survival in IAC patients with a high group (histoscore > 20 in vimentin expression) versus low group (histoscore ≤ 20 in vimentin expression). A two-sided log-rank test was used. **e** The correlation plot of TGR5 with vimentin expression with significant Pearson’s correlation in TCGA LUAD dataset (*n* = 525). R, Pearson’s correlation coefficient. **f**, **g** Transwell migration assays were performed on H1299 cells treated with CA (100 μM), TCDCA (100 μM), and GCDCA (100 μM) or treated with CA (100 μM) and transfected with or without siTGR5. Representative images (left, scale bar, 100 μm) for three biological repeats and statistical analyses (right, *n* = 5) of the migrated cells are shown. siCtrl, siControl. Data represent the mean ± s.e.m. and One-way ANOVA followed by Tukey’s multiple comparison test was used. **h** Diagram showing the experimental design for in vivo metastasis assay (see Methods). **i** Lung metastasis assay of A549-luciferase cells treated with indicated bile acids (Vehicle, *n* = 7; CA, *n* = 8; GCDCA, *n* = 7; TCDCA, *n* = 5, example mice shown to left). **j** Quantification of the metastasis nodules on the pulmonary surface of each groups (Vehicle, *n* = 7; CA, *n* = 8; GCDCA, *n* = 7; TCDCA, *n* = 5). In **i** and **j**, data represent the mean ± s.e.m. and two-tailed Student’s *t*-test was used.

There is increasing evidence that bile acid metabolism plays a role in the progression of various cancers^26–28^. TGR5, G protein-coupled bile acid receptor 1 (GPBAR1), is a bile acid signaling receptor. Activation of TGR5 induced by bile acids leads to an increase in intracellular cyclic AMP (cAMP), thus triggering downstream signaling events that are associated with metabolic disease, inflammation, and cancers^29–31^. In line with the dysregulated bile acid metabolism in S-III subtype, our immunohistochemistry analysis also showed the highest expression of TGR5 in patients from S-III compared to those from S-I and S-II subtype (Fig. 6a, b). Epithelial-to-mesenchymal transition (EMT) is known to be a latent cell-biology programme involved in embryogenesis, wound healing, and malignant cancer progression^32,33^. We found that expression of mesenchymal marker vimentin was also enhanced in S-III compared with S-I and S-II subtype, suggestive of the malignancy of S-III subtype (Fig. 6a, b). Moreover, patients with higher expression of TGR5 or vimentin presented shorter DFS and OS (Fig. 6c, d). Interestingly, analysis of the TCGA LUAD dataset revealed that TGR5 positively correlated with vimentin expression at the mRNA level (Fig. 6e). Together with distinct clinical outcomes in three subtypes, our data supported the potential relevance of TGR5 with LUAD recurrence or metastasis. Given the above findings, we next asked whether dysregulated bile acid metabolism and signaling plays a functional role in the invasive LUAD malignancy. The migration and wound healing assay showed that treatment with the CA, TCDCA, and GCDCA promoted cell migration whereas knockdown of TGR5 suppressed the CA-induced cell migration (Fig. 6f, g and Supplementary Fig. 7a, b). In addition, triamterene, which has recently been used for an antagonist of TGR5^34,35^, blocked the cell migration enhanced by the specific bile acids (Supplementary Fig. 7b), suggesting that TGR5 may be involved in the bile acid-mediated cell migration. To determine whether the cells treated with bile acids would also show heightened metastatic potential in vivo, we pre-treated A549 cells with three kinds of bile acids before injecting them into the tail vein of athymic mice and subcutaneously administrated with bile acids daily at a dose of 3.2 mg kg^−1^ (ref. ^36,37^) (Fig. 6h). Treatment of CA and GCDCA markedly potentiated the ability of the cells to colonize lungs and form metastatic lesions (Fig. 6i, j).

In addition, we also evaluated the effect of manipulating bile acid metabolism and signaling on cell proliferation and found the treatment of CA, TCDCA and GCDCA did not affect cell proliferation and cell survival, however, knockdown of TGR5 significantly inhibited cell survival (Supplementary Fig. 8a–e). Notably, we found that bile acids showed similar levels in EGFR-mutant versus EGFR-WT tumors and neither exogeneous CA, TCDCA, and GCDCA treatment nor knockdown of TGR5 influenced the drug sensitivity to EGFR tyrosine kinase inhibitors (EGFR TKIs), suggesting that bile acid metabolism and signaling may not affect the efficiency of targeted therapy in EGFR-mutant LUAD (Supplementary Fig. 9a–d). Together, these results suggest that perturbed bile acid metabolism and signaling may potentiate migration of LUAD, representing a promising therapeutic target to improve the poor prognosis characteristic of the S-III subtype.

## Discussion

Early detection and intervention contribute to improving clinical outcomes of patients with lung cancer^38^, highlighting an urgent need to gain deep mechanistic insights into the evolutionary trajectory from preneoplasia to invasive LUAD. Genetic and microenvironmental factors have been reported to drive clonal evolution within tumors, which can lead to metabolic liabilities while facilitating cancer progression^39,40^. However, the sophisticated view of how metabolic phenotypes evolve during the neoplastic and invasive progression in LUAD is currently lacking. In this study, we deciphered the metabolic trajectory from AAH to AIS, MIA, and IAC based on a large-scale metabolomics profiling on tissue samples, providing complementary insights beyond the current genomic understanding. We also identified three panels of circulating plasma metabolites to distinguish IAC and its precursors from benign diseases. Our consensus clustering analysis of metabolomics data identified distinct metabolic subtypes that were associated with clinical outcomes, highlighting the potential metabolic vulnerabilities for exploiting precise medicine approaches.

Emerging evidence have demonstrated that metabolic phenotypes evolve during tumor progression from premalignant lesions to locally invasive tumors and metastatic cancer^12^. Recently, gene-expression profiling and multispectral imaging analysis of lung squamous cell carcinoma have identified a transient rise in some metabolic pathways, such as fatty acid metabolism, oxidative phosphorylation, and the citric acid cycle, in preneoplastic lesions^41^. In our study, we observed the gradually altered metabolic pathways, including nicotinate and nicotinamide metabolism, β-Alanine metabolism, glutathione metabolism, arginine and proline metabolism along with the neoplastic and invasive progression of LUAD. Indeed, these metabolic pathways are critical in various cancers^18,42–45^, suggesting that such metabolic vulnerabilities may be exploited for early detection and intervention for LUAD patients. Using c-means clustering, we found that several metabolites showed a specific increase in AIS or in MIA, suggestive of distinct metabolic vulnerabilities in premalignant lesions. Moreover, we also found the progressively upward or downward trend of differential metabolites from AAH to AIS, MIA, and IAC. Specifically, metabolites involved in purine metabolism showed marked increase from the pre-invasive to the invasive stage, implying a dysregulated proliferative state during invasive progression. In line with our findings, a recent study has also demonstrated that the progressive pre-malignant dysplastic lesions are associated with “Proliferative subtypes” which may need close monitoring and early interventions^46^. Overall, integrating clinical and metabolomics data may contribute to scoring systems that are able to identify pre-invasive lesions most likely to progress to malignancy, which may help shape the morphology-based staging criteria. Additionally, therapeutic strategies targeting such key metabolic pathways may play an important role in early intervention for pre-invasive disease, which need to be intensively studied in future work.

Notably, although we delineated the metabolic trajectory from AAH to AIS, MIA, and IAC based on metabolomics data from tissue samples, the metabolomic profiling of plasma samples did not show the evolutionary metabolic changes from preneoplasia to invasive LUAD. In fact, only 16 altered metabolites were identified among four stages from benign diseases to AIS, MIA, and IAC. These findings suggest that cancer progression may not necessarily affect the plasma metabolic signatures systematically. In addition, the correlation between plasma and tumor tissue metabolomics during the disease progression is of great interest, however, we did not observe a clear correlation in this study, probably because the plasma and tissue samples for metabolomics were obtained from different patient cohorts, indicating the potential necessity to collect the plasma and tissue samples from the same cohort for correlation analysis in the future.

The high false-positive rate of radiologic screen in early cancer detection highlights an urgent need for incorporating clinical and morphologic assessment with other non-invasive diagnostics, such as molecular testing and circulating metabolic biomarkers, to enhance the screening efficiency, decrease costs, avoid over-treatment and improve clinical outcomes^21^. However, most current blood-based analyses for diagnosis of LUAD depend on genomic or proteomic biomarkers with finite accuracy^47,48^. Strikingly, our study revealed a biomarker panel of four metabolites was valuable in classifying lung lesions as benign disease or invasive LUAD and its precursors. In addition, a biomarker panel consisting of cystine and valine clearly distinguished benign diseases from premalignant lesions (AIS/MIA), and another biomarker panel consisting of asparagine and cystine was useful in discriminating benign diseases from AIS. Notably, determining whether pulmonary nodules are malignant or benign is critical for clinicians to choose appropriate management. Our findings demonstrate the application of unbiased metabolomics for biomarker discovery, thus serving as an initial cancer screen for the at-risk populations.

Genomics, transcriptomics, and proteomics information have been used to cluster different kinds of cancer types into subgroups^23,49–51^. It is worth noting that a recent study reveals that metabolomics clustering of human clear cell renal cell carcinoma (ccRCC) identifies distinct high-risk and low-risk subtypes^13^. Here, using the data-driven method, we identified three metabolic subtypes which represented distinct metabolic features and correlated with clinical, pathological, and prognostic characteristics of IAC. Interestingly, patients in S-II and S-III subtypes showed more malignancy in clinical and pathological characteristics, such as histological subtypes, TNM stages, tumor size, and smoke history compared to those in S-I. Of note, the relatively large cohort and long clinical follow-up time of our study enable the integration of post-surgical recurrence and/or metastasis as well as overall survival with metabolic subtypes, thus assessing of their prognostic values. This may guide the treatment strategies after surgical resection and aid in the discovery of personalized therapeutic targets. Among the most striking metabolic features, high levels of bile acids identified in S-III correlated with the poor prognosis irrespective of the TNM classification and morphology-based subtypes, which was consistent with previous reports that bile acid metabolism plays an important role in many cancers, such as liver cancer, colorectal cancer, and melanoma metastasis^26,36,52^. These findings suggest that integrating the identification of metabolic biomarkers in biopsy with current clinical classifications may aid in improving the staging criteria more precisely.

The intracellular nuclear receptor farnesoid X receptor (FXR) and membrane receptor TGR5 respond to bile acids by activating transcriptional networks or signaling cascades associated with metabolic disease, inflammation, and cancers^28^. Of note, GCDCA has been reported to promote invasion and migration of liver cancer through AMPK/mTOR-dependent autophagy activation^53^. In addition, another study recently shows that some bioactive bile acids accumulated to high levels in the melanoma metastatic sentinel lymph nodes (LN) activate YAP and fatty acid oxidation, and stimulate further growth of the LN-metastatic tumor^36^. Intriguingly, our study revealed the highest expression of TGR5 and EMT marker vimentin in the S-III subtype and the correlates of high TGR5 or vimentin expression with poor prognosis. Moreover, several bile acids, such as CA, TCDCA, GCDCA, contributed to the enhanced metastatic potential of LUAD. These data indicate that manipulating bile acid metabolism and signaling could be a potential therapeutic strategy in the aggressive subtype of invasive LUAD. Targeted therapy and immunotherapy have led to prodigious survival benefits in selected patients^54^. In our study, bile acids showed similar levels in EGFR-mutant versus EGFR-WT tumors, and manipulation of bile acid metabolism showed no effect on the targeted therapy response using in vitro experiments, suggesting that dysregulated bile acid metabolism may not influence the targeted therapy of LUAD. Notably, it has been well-known that therapeutics targeting metabolic reprogramming, such as low glucose levels, hypoxia, and suppressive metabolites production, show promise as combination therapies for different types of cancer to enhance anticancer immune responses^55–59^. For example, melanoma patients bearing oxidative tumors, which are more hypoxic than glycolytic tumors, show resistance to PD1 blockade through a more exhausted T cell characteristic^60^. In addition, increased arginine levels can shift T cell metabolism from glycolysis to OXPHOS and support its antitumor function^61^. Interestingly, a previous study has demonstrated that gut microbiome use bile acids as messengers to modulate the chemokine-dependent accumulation of hepatic NKT cells and liver antitumor immunosurveillance, and thus suppress both primary and metastatic tumors^26,62^, indicating the importance of bile acid metabolism in regulating the tumor immune microenvironment. Therefore, it is interesting to explore the effect of aberrant bile acid metabolism on immunotherapy in LUAD in the future.

In summary, the metabolomics analysis of patients with different stages deciphers evolutionary metabolic trajectory from preneoplasia to invasive LUAD, thus providing a comprehensive understanding of the neoplastic and invasive progression of LUAD and further offering an opportunity to expedite the translation of basic research to more precise diagnosis and therapy in the clinic.

## Methods

### Clinical samples

There are two clinical cohorts collected for tumor tissue and plasma metabolomics analysis. The patient tissue specimens for this study were collected from Shanghai Pulmonary Hospital during December 2015 to June 2016. Briefly, a total of 181 patients were enrolled in this study, including 12 AAH, 22 AIS, 19 MIA, and 128 IAC patients. Patients receiving any anti-cancer treatments before surgery were excluded. Primary tumor tissues and paired non-cancerous adjacent normal tissues (>3 cm apart from tumor edge) were surgically resected and transferred to liquid nitrogen during operation and stored in −80 °C refrigerators until being used for metabolite extraction. Clinical information including gender, age, smoke history, tumor size, histological subtype, TNM stage, the status of recurrence, and status of survival were collected. The plasma samples were collected from a total of 92 patients in Shanghai Pulmonary Hospital, which contained 10 patients with benign diseases, 32 patients with AIS, 22 patients with MIA, and 28 patients with IAC. The benign diseases included inflammatory lesions, granuloma, lymphoid hyperplasia, bronchiolal metaplasia, and fibrosis. The plasma collection criteria for all patients were as follows: fasted at least 6 h and blood was drawn in the morning using BD Vacutainer EDTA tubes. The blood was then centrifuged at 1000 × g for 10 min at 4 °C. The plasma supernatant was collected and centrifuged at 2000 × g for 5 min at 4 °C. After aliquoting, plasma was frozen at −80 °C until metabolite extraction. Detailed characteristics of the patient populations were provided in Supplementary Data 1. The study was approved by the Research Ethics Committee of Shanghai Pulmonary Hospital (Institutional Review Board: K20-317), and written informed consent was obtained from each patient.

### Metabolite extraction

For metabolite extraction of tissue samples, each tissue sample was accurately weighed and homogenized in an ice-cold 80% methanol aqueous solution (50 mg tissue/mL). 100 µL of homogeneous tissue sample was mixed with 900 µL ice-cold 80% methanol aqueous solution. The mixture was then vortexed and centrifuged at 15,000 × g for 15 min at 4 °C. 900 µL supernatant was then transferred to a new tube. The pellet was mixed with 500 µL ice-cold 80% methanol and re-centrifuged at 15,000 × g for 15 min at 4 °C, 500 µL supernatant was collected and combined with the previous supernatant. After being divided into 2 equal aliquots, the supernatant was dried under a speed vacuum concentrator. The dried metabolite pellets were kept at −80 °C until LC-MS analysis. Quality control (QC) samples were prepared by pooling 10 µL homogenate of each tissue sample. Pretreatment of QC samples was paralleled and the same to the study samples. For metabolite extraction of plasma samples, 80 µL plasma of each patient was mixed with 320 µL ice cold methanol. The mixture was then vortexed and centrifuged at 15,000 × g for 15 min at 4 °C. The supernatant was divided into 3 replicates and evaporated to dryness under a speed vacuum concentrator. For QC sample preparation, 20 µL plasma of each patient was mixed and then processed the same as that of the study plasma samples.

### Targeted metabolomics

For targeted metabolomics, all tissue samples from 181 patients were randomly divided into 14 analytical batches, with paired samples from each patient included and randomized. QC samples were evenly inserted in each analytical batch to monitor the instrumentation stability and subsequently used to correct inter-batch effects. Similarly, 92 plasma samples were randomly analyzed in 2 analytical batches with QC samples evenly inserted.

Dried metabolites were reconstituted in 50 μL of 0.03% formic acid in analytical-grade water, vortex-mixed, and centrifuged to remove debris. Thereafter, the supernatant was transferred to a high-performance liquid chromatography (HPLC) vial for the metabolomics study. Targeted metabolite profiling was performed using a liquid chromatography-mass spectrometry/mass spectrometry (LC–MS/MS) approach. Chromatographic separation was achieved on an ACQUITY UPLC HSS T3 column (2.1 × 150 mm, 1.8 µm) using an ACQUITY UPLC I-Class system (Waters). The mobile phases employed were 0.03% formic acid in water (A) and 0.03% formic acid in acetonitrile (B) and the gradient program was as follows: 0–3 min, 1% B; 3–15 min, 1%–99% B; 15–17 min, 99% B; 17–17.1 min, 99% - 1% B; 17.1–20 min, 1% B. The column was maintained at 40 °C and the samples were kept in the autosampler at 4 °C. The flow rate was 0.25 mL/min, and the injection volume was 10 µL. A Xevo TQ-XS mass spectrometer (Waters) with electrospray ionization (ESI) source was operated in multiple reaction monitoring (MRM) mode for mass data acquisition. The MS parameters were set as follows: Capillary voltage 1.0 kV (positive) and 2.5 kV (negative), desolvation temperature 450 °C, desolvation gas flow 900 L/Hr. Cone voltage (CV) and collision energy (CE) were optimized for each metabolite by direct infusion of reference standards using a syringe pump prior to sample analysis. With positive and negative switching mode, a total of 261 metabolites were monitored including 165 ion transitions in positive mode and 96 ion transitions in negative mode. Metabolite classification was gathered from HMDB (http://hmdb.ca).

### Metabolomics data analysis

Chromatogram review and peak area integration were performed using Skyline 4.1 software (MacCoss Lab). The processed data were exported for further analysis. The missing value was removed according to the 80% rule^63^, wherein, a metabolite was considered as detectable when it was detected across at least 4/5 samples in one group, and undetected metabolites (<1/5 samples) were filled with a detection baseline value, 1000, to allow the following statistical analysis.

To remove potential inter-batch variations, the mean peak area of each metabolite from all the QC samples in all given batches (QC_all_), as well as the mean peak area of each metabolite from the QC samples that are the most adjacent to a given group of test samples (QC_adj_) were first calculated. The ratio between these two mean peak areas for each metabolite was computed by dividing the same QC_all_ by each QC_adj_ and used as the normalization factor for each given group of test samples. The peak area of each metabolite from each test sample were normalized by multiplying their corresponding normalization ratio to obtain the normalized peak areas. In addition, to effectively correct the sample to sample variation in biomass that may contribute to systematic differences in metabolite abundance detected by LC-MS, we generated the scaled data by comparing the normalized peak area of each metabolite to the sum of the normalized peak area from all the detected metabolites in that given sample.

Relative abundance was defined as the ratio of metabolite intensity (tumor/NAT) of each patient, which was used to further analyze metabolic differences between various stages of lung adenocarcinoma. The multivariate analyses, including principal component analysis (PCA) and partial least squares discrimination analysis (PLS-DA), were carried out using SIMCA-P (Umetrics, Umea, Sweden), prcomp, or plsda function in the R stat package. The correlation of metabolites with disease progression was evaluated using Mfuzz (v.2.46.0) R package on the metabolomics data.

### Logistic regression analysis

The circulating metabolite biomarker model for predicting disease stages was established and evaluated using SPSS software (v.27.0). Binary logistic regression was used to identify predictive factors associated with tumor progression. A receiver-operating characteristics curve was used to evaluate the results of the regression and analysis.

### Bile acid detection

The tumor and paired NAT obtained from 126 IAC patients were used for bile acid detection. The dried metabolite extracts were reconstituted in 30 μL of 10% acetonitrile in water, vortexed, centrifuged at 15,000 × g for 15 min at 4 °C and the supernatant was analyzed using LC–MS/MS. Detection of 9 bile acids was performed according to the previously described method with modification^64^. Briefly, an Ultra High Performance Liquid Chromatograph (UHPLC) system (Nexera × 2 LC-30A, Shimadzu) was used for metabolites separation, with an ACQUITY UPLC HSS-T3 UPLC column (50 × 2.1 mm, 1.8 μm, Waters) and the following gradient: 0–0.3 min 10% mobile phase B, 0.3–1.9 min 10–50% B; 1.9–2.1 min 50% B; 2.1–4.0 min 50–95% B; 4.0–4.9 min 95% B; 4.9–5.0 min 95–10% B; 5.0–5.5 min 10% B. Mobile phase A was 0.1% formic acid in water. Mobile phase B was 0.1% formic acid in acetonitrile. The flow rate was 0.7 mL/min, the column was kept at 60 °C and the samples in the autosampler were at 4 °C. The injection volume was 20 μL. Mass spectrometry analysis was performed with a triple quadrupole mass spectrometer (Qtrap 6500+, SCIEX) under negative mode with MRM mode. The MRM transitions for CA, CDCA, DCA, GCA, GCDCA, GDCA, TCA, TCDCA, TDCA were 407.2 > 407.2, 391.3 > 391.3, 391.3 > 391.3, 464.3 > 74.1, 448.4 > 73.9, 448.3 > 74.2, 514.2 > 80.1, 498.3 > 80, and 498.2 > 80, respectively. The data were acquired using Analyst Instrument Control software v.1.6.3 (SCIEX) and data processing was performed using MultiQuant software v.3.0 (SCIEX). The ratio of bile acid abundance (tumor/NAT) of each patient was calculated for further analysis.

### Metabolomics consensus clustering

Unsupervised clustering of the metabolomics data of IAC patient tumor tissue samples was performed using R package ‘ConsensusClusterPlus’ (v1.50.0)^65^ to generate subtypes. Patient clusters were derived based on k-means clustering, Pearson distance, and 1,000 resampling repetitions in the range of 2 to 6 clusters. The number of clustering was determined by two factors: the average pairwise consensus matrix within consensus clusters, and the delta plot of the relative change in the area under the cumulative distribution function (CDF) curve. Based on the evidence above, the metabolomics data of IAC patients were clustered into three subtypes.

### Pathway enrichment analysis

KEGG pathway enrichment analysis based on significantly differential metabolites between the indicated sample groups, including the histological subtypes, paired tumor/NAT group, and metabolomics subtypes, was assessed by R package ‘clusterProfiler’ (v3.14.3). Fold enrichment and mean log2 fold change of significant metabolites in each pathway were calculated. KEGG metabolic pathways and related metabolites were downloaded through KEGG API (https://www.kegg.jp/kegg/rest/keggapi.html). Significant enriched KEGG pathways were determined with Fisher’s exact test followed by BH multiple comparison test with FDR < 0.05. TCGA dataset of patients with LUAD was obtained from the GDC database (https://xenabrowser.net/datapages/). Another transcriptomics dataset of patients with LUAD was obtained from the published study^23^. Differential Expressed Genes (DEGs) were identified using R package ‘edgeR’ (v3.28.1)^66^. We selected the genes which had FDR < 0.05 and fold change > 1.5 or <0.67. The DEGs between tumor and NAT of LUAD were used for KEGG pathway enrichment analysis by R package ‘clusterProfiler’ (v3.14.3) and pathways with FDR < 0.1 were selected. Proteomics data of patients with LUAD were obtained from the published study^23^. KEGG pathway enrichment analysis was performed on the reported differential proteins using the R package ‘clusterProfiler’ (v3.14.3) and pathways with FDR < 0.1 were selected.

### Association between metabolomics data and clinical outcome

Survival analysis was performed using the R package ‘survival’ (v3.2-11). For the association analysis between metabolite level with survival, Kaplan-Meier survival curves (log-rank test) were used to compare overall survival or disease-free survival outcomes among two groups. Kaplan-Meier survival curves (log-rank test) were used to compare overall survival or disease-free survival outcomes among three subtypes. Kaplan-Meier survival curves were plotted by function ggsurvplot in R package ‘survminer’ (v0.4.9). For correlation analysis between metabolomic subtypes and clinical features, we performed Fisher’s exact test on categorical variables, including age, tumor size, histological types, stage, smoke status, and metastasis/recurrence status.

### Cell culture and siRNA transfection

H1299, A549, HCC827, and H1975 cells were purchased from the ATCC and cultured in Roswell Park Memorial Institute (RPMI) 1640 medium supplemented with 10% fetal bovine serum (FBS). All cell lines were tested negative for mycoplasma. For siRNA transfection, lipofectamine RNAiMAX (Thermo) was used according to the standard protocol. All siRNAs targeting the genes were obtained from Genepharm Technologies. The two TGR5 siRNA targeting sequences were listed in the Supplementary Table 1.

### Western blot analysis

Cells were homogenized in RIPA butter supplemented with protease and phosphatase inhibitors using a cell scraper. Lysates were centrifuged at 15,000 × g for 15 min at 4 °C. Protein concentration was quantified using a bicinchoninic acid (BCA) assay. A total of 30 µg from each sample were loaded into each lane and separated by electrophoresis on a 10% SDS polyacrylamide gel. After electrophoresis, proteins were transferred to cellulose nitrate membranes. Nonspecific binding was blocked through incubation with PBST (PBS with 0.1% Tween 20) containing 5% skim milk for at least 1 h at room temperature. Membranes were incubated with antibodies including TGR5 (1:1000, Abcam, catalog No: ab72608) and α-Tubulin (1:3000, ABclonal, catalog No: AC012). For detection, HRP-linked anti-rabbit and anti-mouse IgG secondary antibodies were used and a chemiluminescent signal was detected with a digital imager (Biotanon, Tanon 5200).

### Cell proliferation and viability assay

In brief, 2.5 × 10^4^ cells/well were seeded in 6-well plates and treated with or without exogenous bile acids for indicated time points. Cell numbers were counted with the thermo Countess II. Cell viability assays were conducted with CellTiter Glo reagent (Promega) following the manufacturer’s protocol. Cells were cultured in 96-well plates with the treatment of indicated compounds for 48 h or 72 h.

### Wound healing and migration assay

For the wound healing assay, 3 × 10^5^ cells/well were seeded in 6-well plates and incubated with indicated compounds. The compounds including CA (S3742), TCDCA (S3865), GCDCA (S5794), and triamterene (S4080) were purchased from Selleck. Monolayers were scratched with the same pipette tip. The wound closure was monitored 48 h after scratch and three random fields of the view were recorded. For the transwell migration assay, approximately 4 × 10^4^ cells under different conditions were resuspended in a 200 µL medium containing 2% FBS and then placed onto the upper chamber of a transwell filter with 8-µm pores. The 600 µL medium containing 10% FBS was added to the lower chamber. After 48 h, cells on the underside of the filter were fixed with 4% paraformaldehyde in PBS and were stained with 0.4% crystal violet in 10% ethanol. The migrated cells in five random fields were counted and imaged.

### Lung metastasis assay in mice

All procedures involving mice and experimental protocols were approved by the Institutional Animal Care and Use Committee of Tsinghua University. Luciferase-expressing A549 cells were pre-treated with 100 μM CA, GCDCA, and TCDCA for 48 h before injection via the tail vein for evaluation of lung colonization. In brief, 8-week-old female nu/nu athymic mice were injected with 4 × 10^6^ cells in 200 μL of PBS per mouse. Successful injections were confirmed by bioluminescence imaging immediately after injection. After 5 days, mice were injected with or without CA, GCDCA, and TCDCA (3.2 mg per kg, daily) subcutaneously. Metastases were monitored using IVIS Spectrum In Vivo Imaging System (Perkin-Elmer). 4 weeks after injection, luminescence was measured and quantified using IVIS-Image Software v.4.3.1 (Perkin-Elmer) to determine lung colonization.

### Immunohistochemistry

The formalin-fixed and paraffin-embedded specimens obtained from 126 IAC patients were prepared and provided by the Pathology Department of Shanghai Pulmonary Hospital. Slides were stained with TGR5 antibody (1:200, Abcam, catalog No: ab72608) and vimentin antibody (1:2000, Proteintech, catalog No: 10366-1-AP), and were processed using the standard procedures. For scoring the IHC image, histoscore was calculated by the proportion of positive cells of tumor tissue (0–100%) by the average intensity of the positive staining (negative staining as 0, weak staining as 1, moderate staining as 2, and strong staining as 3), so as to obtain the score ranging from 1 to 300 for each sample. The hematoxylin-eosin slides and the immunohistochemistry slides were examined and evaluated independently by two experienced pathologists. The histoscore was calculated as the average scores obtained from the two pathologists. Histoscore was compared with the disease-free survival and overall survival. Disease-free survival was defined as the date from the date of diagnosis to recurrence, overall survival was defined as the time from the date of diagnosis to death. To separate patients into two groups, the optimal cutoff value was determined using the surv_cutpoint function of the R package survminer (v0.4.9). The Kaplan-Meier method was used to generate survival curves by ggsurvplot function of the R package survminer (v0.4.9) and the significance of differences was compared using the log-rank test.

### Statistical analysis

Methods of quantification and statistical analysis for metabolomic analyses and all experiments were described in the Results, figure legends, and corresponding Method subsections. The sample distribution was determined by the Shapiro-Wilk test normality test and quantile-quantile plot (Q-Q plot). Standard statistical tests were used to analyze the clinical data. Specifically, two-sided Wilcoxon rank-sum test and Wilcoxon signed-rank test were used when comparing two groups for unpaired samples and paired samples, respectively. A two-sided Kruskal–Wallis test was used when comparing three or more groups. *P* values were corrected for multiple testing using the Benjamini-Hochberg procedure. Either GraphPad Prism or R were used to conduct tests.

### Reporting summary

Further information on research design is available in the Nature Research Reporting Summary linked to this article.

### Supplementary information

Supplementary Information

Description of Additional Supplementary Files

Supplementary Data 1

Supplementary Data 2

Supplementary Data 3

Reporting summary

### Source data

Source Data

## Data Availability

Clinical information of patients with different stages is included in Supplementary Data1. Raw metabolomics mass spectrometry data are included in Supplementary Data 2. Normalized metabolomics data are included in Supplementary Data 3. The GDC TCGA LUAD dataset can be downloaded from https://xenabrowser.net/datapages/. The previously published^23^ RNA-seq data and proteomics data can be obtained from 10.1016/j.cell.2020.05.043. Raw data for plotting figures are provided in the Source Data file. Source data are provided with this paper.

## Code availability

Code for processing metabolomics data is available at https://github.com/iekoay/LUAD_NCOMMS

## References

[1] Bray F (2018). Global cancer statistics 2018: GLOBOCAN estimates of incidence and mortality worldwide for 36 cancers in 185 countries. CA Cancer J. Clin..

[2] Chen Z, Fillmore CM, Hammerman PS, Kim CF, Wong KK (2014). Non-small-cell lung cancers: a heterogeneous set of diseases. Nat. Rev. Cancer.

[3] Weichert W, Warth A (2014). Early lung cancer with lepidic pattern: adenocarcinoma in situ, minimally invasive adenocarcinoma, and lepidic predominant adenocarcinoma. Curr. Opin. Pulm. Med..

[4] National Lung Screening Trial Research, T. (2011). Reduced lung-cancer mortality with low-dose computed tomographic screening. N. Engl. J. Med..

[5] Hu X (2019). Multi-region exome sequencing reveals genomic evolution from preneoplasia to lung adenocarcinoma. Nat. Commun..

[6] Chen H (2019). Genomic and immune profiling of pre-invasive lung adenocarcinoma. Nat. Commun..

[7] Zhang C (2019). Genomic landscape and immune microenvironment features of preinvasive and early invasive lung adenocarcinoma. J. Thorac. Oncol..

[8] Devarakonda S, Govindan R (2019). Untangling the evolutionary roots of lung cancer. Nat. Commun..

[9] Xing, X. et al. Decoding the multicellular ecosystem of lung adenocarcinoma manifested as pulmonary subsolid nodules by single-cell RNA sequencing. *Sci Adv* **7**, 10.1126/sciadv.abd9738 (2021). 10.1126/sciadv.abd9738 PMC7840134 33571124

[10] Dejima H (2021). Immune evolution from preneoplasia to invasive lung adenocarcinomas and underlying molecular features. Nat. Commun..

[11] Keibler MA (2016). Metabolic requirements for cancer cell proliferation. Cancer Metab..

[12] Faubert, B., Solmonson, A. & DeBerardinis, R. J. Metabolic reprogramming and cancer progression. *Science* **368**, 10.1126/science.aaw5473 (2020). 10.1126/science.aaw5473 PMC7227780 32273439

[13] Hakimi AA (2016). An integrated metabolic atlas of clear cell renal cell carcinoma. Cancer Cell.

[14] Yachida S (2019). Metagenomic and metabolomic analyses reveal distinct stage-specific phenotypes of the gut microbiota in colorectal cancer. Nat. Med..

[15] Scafoglio, C. R. et al. Sodium-glucose transporter 2 is a diagnostic and therapeutic target for early-stage lung adenocarcinoma. *Sci Transl Med* **10**, 10.1126/scitranslmed.aat5933 (2018). 10.1126/scitranslmed.aat5933 PMC6428683 30429355

[16] Wang Z (2019). Methionine is a metabolic dependency of tumor-initiating cells. Nat. Med..

[17] Locasale JW (2013). Serine, glycine and one-carbon units: cancer metabolism in full circle. Nat. Rev. Cancer.

[18] Sanderson SM, Gao X, Dai Z, Locasale JW (2019). Methionine metabolism in health and cancer: a nexus of diet and precision medicine. Nat. Rev. Cancer.

[19] National Lung Screening Trial Research, T. (2013). Results of initial low-dose computed tomographic screening for lung cancer. N. Engl. J. Med.

[20] de Koning HJ (2020). Reduced lung-cancer mortality with volume CT screening in a randomized trial. N. Engl. J. Med..

[21] Cohen JD (2018). Detection and localization of surgically resectable cancers with a multi-analyte blood test. Science.

[22] Vanhove K (2018). The plasma glutamate concentration as a complementary tool to differentiate benign PET-positive lung lesions from lung cancer. BMC Cancer.

[23] Xu JY (2020). Integrative proteomic characterization of human lung adenocarcinoma. Cell.

[24] Chen YJ (2020). Proteogenomics of non-smoking lung cancer in east asia delineates molecular signatures of pathogenesis and progression. Cell.

[25] Gillette MA (2020). Proteogenomic characterization reveals therapeutic vulnerabilities in lung adenocarcinoma. Cell.

[26] Ma, C. et al. Gut microbiome-mediated bile acid metabolism regulates liver cancer via NKT cells. *Science* **360**, 10.1126/science.aan5931 (2018). 10.1126/science.aan5931 PMC6407885 29798856

[27] Dart A (2018). Gut microbiota bile acid metabolism controls cancer immunosurveillance. Nat. Rev. Microbiol.

[28] Jia W, Xie G, Jia W (2018). Bile acid-microbiota crosstalk in gastrointestinal inflammation and carcinogenesis. Nat. Rev. Gastroenterol. Hepatol..

[29] Guo C, Chen WD, Wang YD (2016). TGR5, not only a metabolic regulator. Front Physiol..

[30] Stepanov V, Stankov K, Mikov M (2013). The bile acid membrane receptor TGR5: a novel pharmacological target in metabolic, inflammatory and neoplastic disorders. J. Recept Signal Transduct. Res..

[31] Zhao RY (2018). High expression of TGR5 predicts a poor prognosis in patients with pancreatic cancer. Int J. Clin. Exp. Pathol..

[32] Lambert AW, Weinberg RA (2021). Linking EMT programmes to normal and neoplastic epithelial stem cells. Nat. Rev. Cancer.

[33] Dongre A, Weinberg RA (2019). New insights into the mechanisms of epithelial-mesenchymal transition and implications for cancer. Nat. Rev. Mol. Cell Biol..

[34] Li Y (2017). Investigation of triamterene as an inhibitor of the TGR5 receptor: identification in cells and animals. Drug Des. Devel Ther..

[35] Qi X (2019). Gut microbiota-bile acid-interleukin-22 axis orchestrates polycystic ovary syndrome. Nat. Med.

[36] Lee CK (2019). Tumor metastasis to lymph nodes requires YAP-dependent metabolic adaptation. Science.

[37] Nie B (2012). Specific bile acids inhibit hepatic fatty acid uptake in mice. Hepatology.

[38] Goldberg SW, Mulshine JL, Hagstrom D, Pyenson BS (2010). An actuarial approach to comparing early stage and late stage lung cancer mortality and survival. Popul Health Manag.

[39] Yuneva MO (2012). The metabolic profile of tumors depends on both the responsible genetic lesion and tissue type. Cell Metab..

[40] Hensley CT (2016). Metabolic heterogeneity in human lung tumors. Cell.

[41] Mascaux C (2019). Immune evasion before tumour invasion in early lung squamous carcinogenesis. Nature.

[42] Gao X (2019). Dietary methionine influences therapy in mouse cancer models and alters human metabolism. Nature.

[43] Triantafyllou EA, Georgatsou E, Mylonis I, Simos G, Paraskeva E (2018). Expression of AGPAT2, an enzyme involved in the glycerophospholipid/triacylglycerol biosynthesis pathway, is directly regulated by HIF-1 and promotes survival and etoposide resistance of cancer cells under hypoxia. Biochim Biophys. Acta Mol. Cell Biol. Lipids.

[44] Dolce V, Cappello AR, Lappano R, Maggiolini M (2011). Glycerophospholipid synthesis as a novel drug target against cancer. Curr. Mol. Pharm..

[45] Shukla SK (2017). MUC1 and HIF-1alpha signaling crosstalk induces anabolic glucose metabolism to impart gemcitabine resistance to pancreatic cancer. Cancer Cell.

[46] Beane JE (2019). Molecular subtyping reveals immune alterations associated with progression of bronchial premalignant lesions. Nat. Commun..

[47] Chabon JJ (2020). Integrating genomic features for non-invasive early lung cancer detection. Nature.

[48] Geary B (2019). Identification of a biomarker panel for early detection of lung cancer patients. J. Proteome Res.

[49] Xiong J (2020). Genomic and transcriptomic characterization of natural killer T cell lymphoma. Cancer Cell.

[50] Gao Q (2019). Integrated proteogenomic characterization of HBV-related hepatocellular carcinoma. Cell.

[51] Vasaikar S (2019). Proteogenomic analysis of human colon cancer reveals new therapeutic opportunities. Cell.

[52] Fu T (2019). FXR regulates intestinal cancer stem cell proliferation. Cell.

[53] Gao L (2019). Glycochenodeoxycholate promotes hepatocellular carcinoma invasion and migration by AMPK/mTOR dependent autophagy activation. Cancer Lett..

[54] Herbst RS, Morgensztern D, Boshoff C (2018). The biology and management of non-small cell lung cancer. Nature.

[55] Jayaprakash P (2018). Targeted hypoxia reduction restores T cell infiltration and sensitizes prostate cancer to immunotherapy. J. Clin. Invest.

[56] Leone RD (2019). Glutamine blockade induces divergent metabolic programs to overcome tumor immune evasion. Science.

[57] Sukumar M (2013). Inhibiting glycolytic metabolism enhances CD8+ T cell memory and antitumor function. J. Clin. Invest.

[58] DeBerardinis RJ (2020). Tumor microenvironment, metabolism, and immunotherapy. N. Engl. J. Med.

[59] DePeaux, K. & Delgoffe, G. M. Metabolic barriers to cancer immunotherapy. *Nat Rev Immunol*, 10.1038/s41577-021-00541-y (2021). 10.1038/s41577-021-00541-y PMC8553800 33927375

[60] Najjar, Y. G. et al. Tumor cell oxidative metabolism as a barrier to PD-1 blockade immunotherapy in melanoma. *JCI Insight* **4**, 10.1172/jci.insight.124989 (2019). 10.1172/jci.insight.124989 PMC6483505 30721155

[61] Geiger R (2016). L-arginine modulates T cell metabolism and enhances survival and anti-tumor activity. Cell.

[62] Schramm C (2018). Bile acids, the microbiome, immunity, and liver tumors. N. Engl. J. Med.

[63] Bijlsma S (2006). Large-scale human metabolomics studies: a strategy for data (pre-) processing and validation. Anal. Chem..

[64] Zheng JJ (2016). The utility of stable isotope labeled (SIL) analogues in the bioanalysis of endogenous compounds by LC-MS applied to the study of bile acids in a metabolomics assay. Anal. Biochem.

[65] Wilkerson MD, Hayes DN (2010). ConsensusClusterPlus: a class discovery tool with confidence assessments and item tracking. Bioinformatics.

[66] Robinson MD, McCarthy DJ, Smyth GK (2010). edgeR: a Bioconductor package for differential expression analysis of digital gene expression data. Bioinformatics.

