# Supplementary material for: Evolutionary metabolic landscape from preneoplasia to invasive lung adenocarcinoma
Source: Nat Commun. 2021 Nov 10;12:6479. doi: 10.1038/s41467-021-26685-y (PMC8580984; doi:10.1038/s41467-021-26685-y)
Supplement: Supplementary file 1 — Supplementary Information [file 41467_2021_26685_MOESM1_ESM.pdf]

Supplementary Information

Evolutionary metabolic landscape from preneoplasia to invasive lung adenocarcinoma

Authors

Meng Nie[1,6], Ke Yao[1,6], Xinsheng Zhu[2,6], Na Chen[1,6], Nan Xiao[1], Yi Wang[1], Bo Peng[1], LiAng Yao[1], Peng Li[3,4,5], Peng Zhang[2,*], and Zeping Hu[1,*]

Affiliations

[1]School of Pharmaceutical Sciences, Tsinghua-Peking Center for Life Sciences, Beijing Frontier Research Center for Biological Structure, Tsinghua University, Beijing, 100084, China.

[2]Department of Thoracic Surgery, Shanghai Pulmonary Hospital, Tongji University School of Medicine, Shanghai, 200433, China.

[3]Institute of Metabolism and Integrative Biology, Fudan University, Shanghai, 200438, China

[4]Shanghai Qi Zhi Institute, Shanghai, 200030, China

[5]School of Life Sciences, Tsinghua University, Beijing 100084, China

[6]These authors contributed equally

***Correspondence:**

zhangpeng1121@tongji.edu.cn (P.Z.), zeping_hu@tsinghua.edu.cn (Z.H.)

Supplementary Figure 1

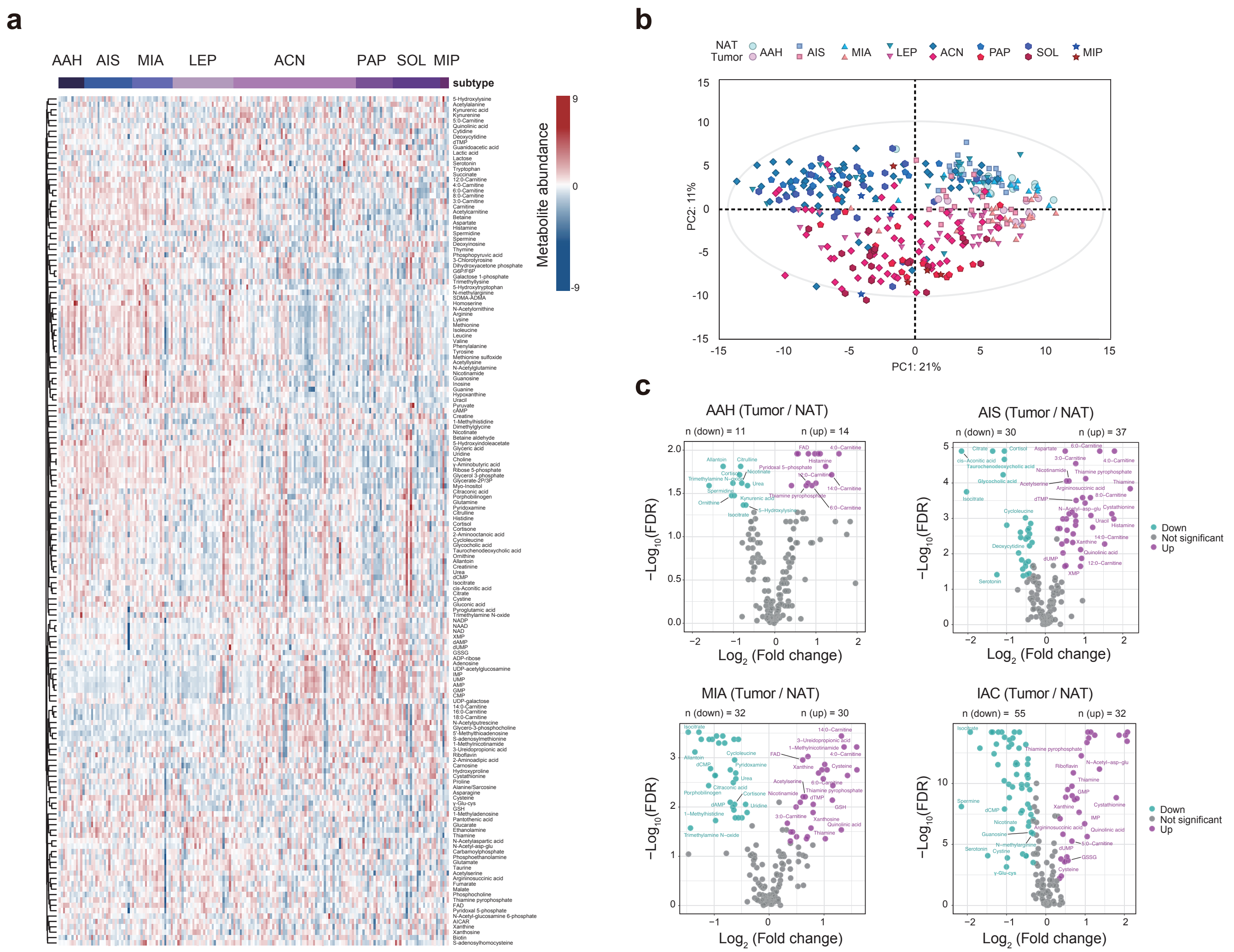

Supplementary Figure 1. Targeted metabolomics from lung preneoplasia to adenocarcinoma.
a, Heatmap comparing 158 profiled metabolites in different morphological stages from lung preneoplasia to invasive adenocarcinoma.
b, Principal component analysis (PCA) of tumor tissue (red) and paired non-cancerous adjacent tissue (NAT, blue) in different morphological stages from lung preneoplasia to invasive adenocarcinoma based on metabolomics data.
c, Volcano plots of the significantly differential metabolites (FDR < 0.05 and fold change > 1.25 or < 0.8) in tumor versus NAT of each morphological stage are shown. Two-sided Wilcoxon signed-rank test followed by Benjamini-Hochberg (BH) multiple comparison test was used. Metabolites significantly increased or decreased in tumor are colored in purple and green, respectively.

Supplementary Figure 2

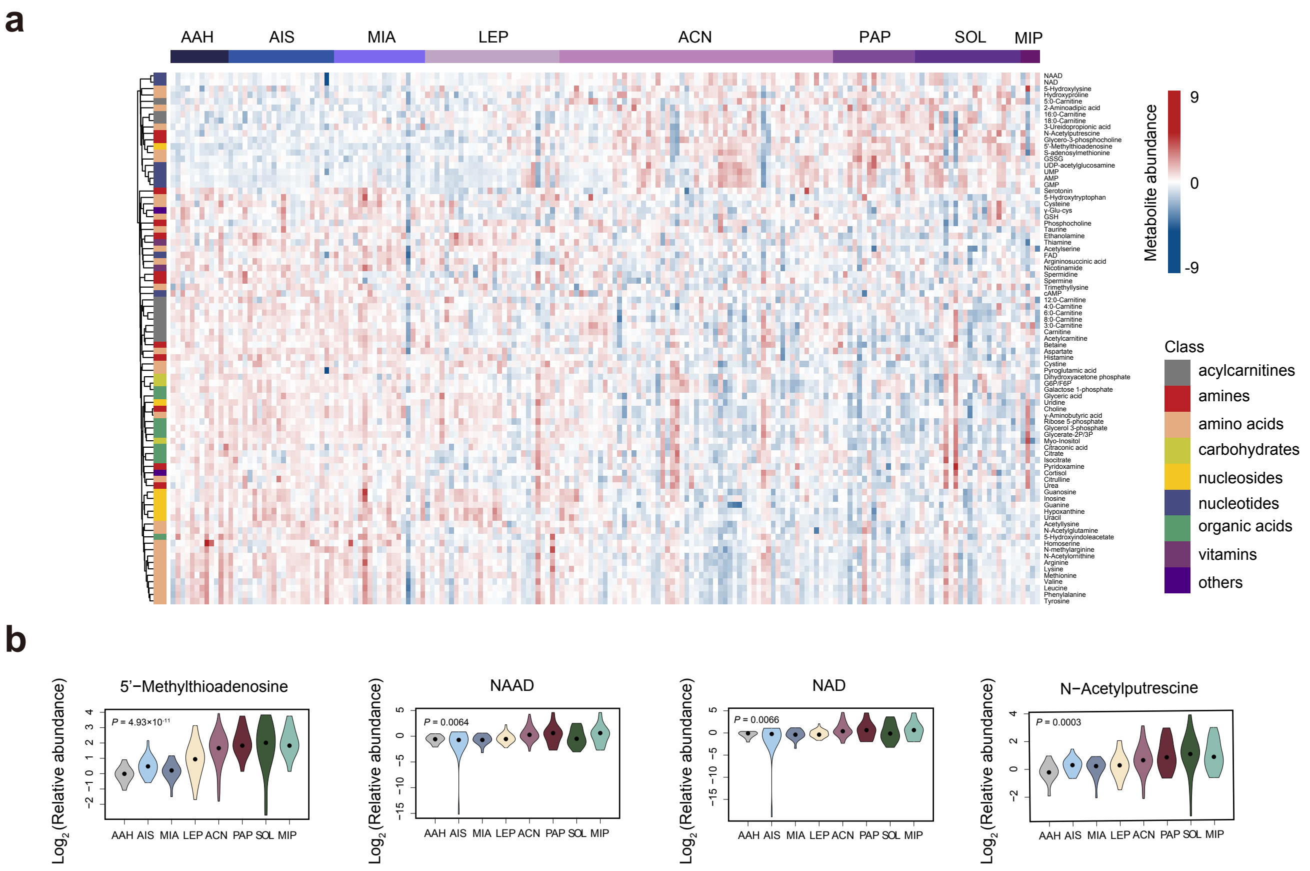

Supplementary Figure 2. Metabolic alterations from AAH to IAC.
a. Heatmap of differential metabolites (FDR < 0.1) among AAH, AIS, MIA and IAC. Classes of metabolites are color-coded. Two-sided Kruskal-Wallis tests followed by Benjamini-Hochberg (BH) multiple comparison test was used.
b. Violin plots of differential metabolites among AAH, AIS, MIA and IAC. Two-sided Kruskal-Wallis test was used. Black dots represent population medians.

Supplementary Figure 3

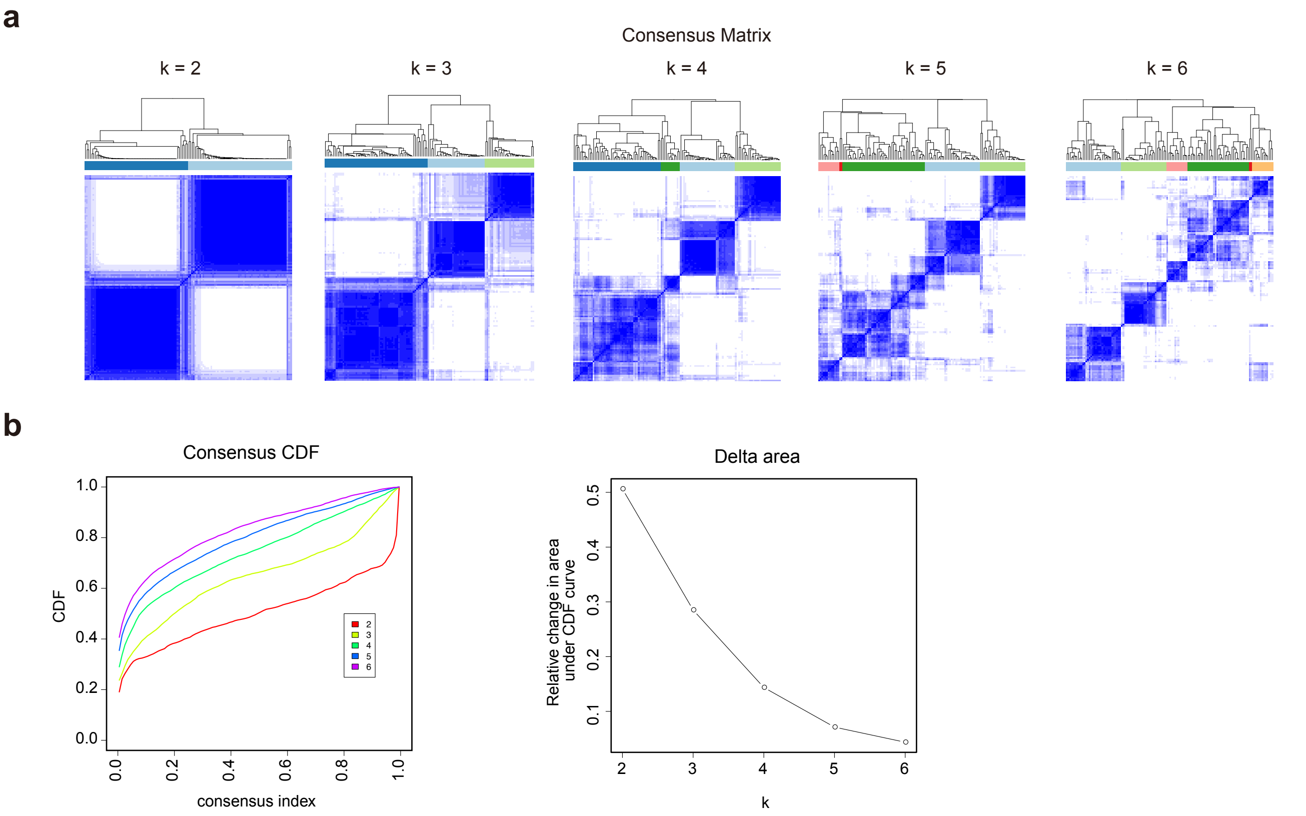

Supplementary Figure 3. Consensus clustering for metabolomics data in the IAC patients.
a. Subtypes are identified based on metabolomics data of IAC patients. k was tested from 2 to 6.
b. Consensus cumulative distribution function (CDF) plot and delta area (change in CDF area) plot are shown.

Supplementary Figure 4

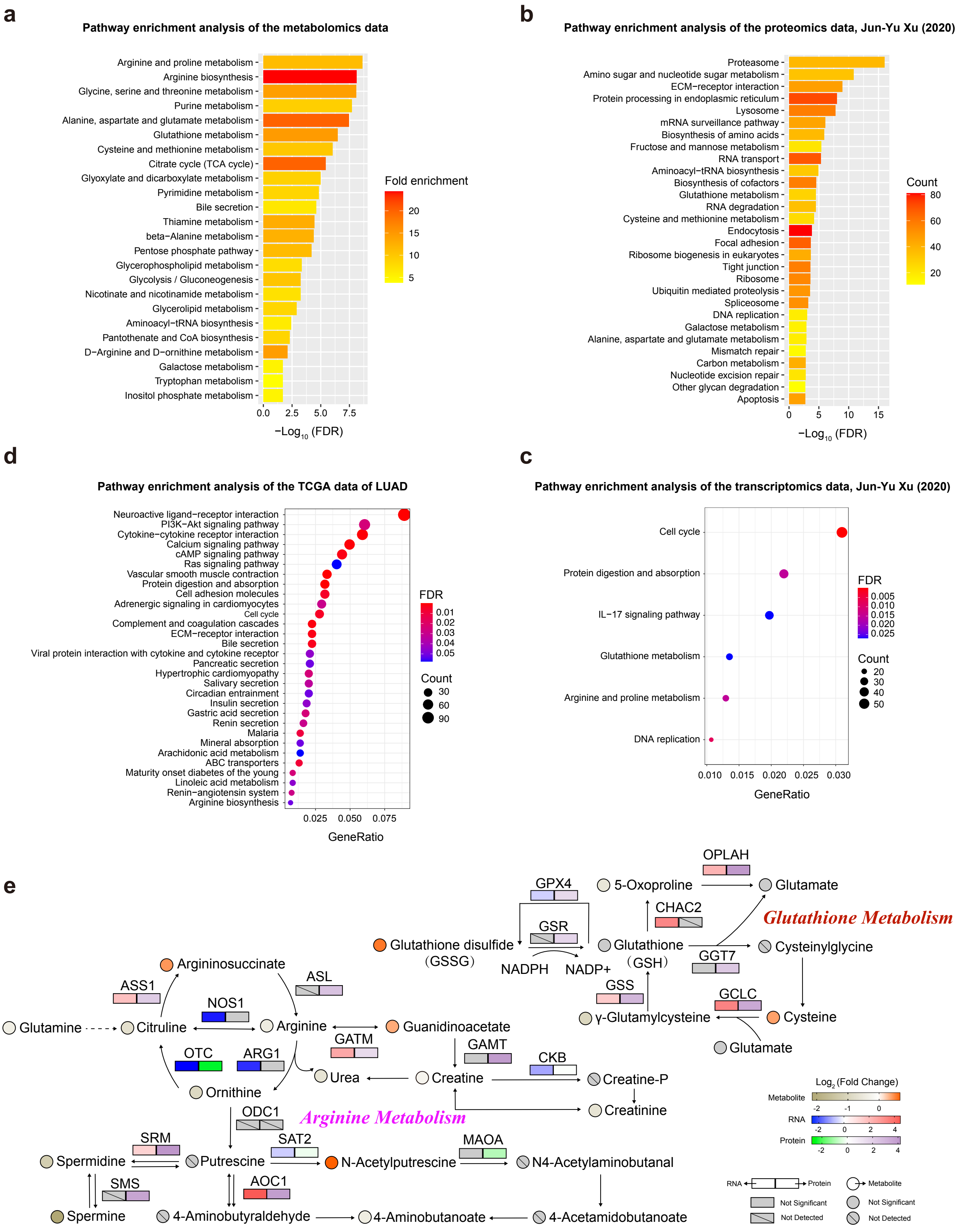

Supplementary Figure 4. Metabolic-pathway based integration of transcriptomics, metabolomics and proteomics data derived from different dataset.

a. KEGG pathway enrichment analysis based in differential metabolites in tumor samples versus NAT of IAC patients.

b, c. KEGG pathway enrichment analysis based in differential proteins (**b**) and differential genes (**c**) in tumor samples versus NAT of LUAD patients. Proteomics and transcriptomics data were obtained from indicated published study.

d. KEGG pathway enrichment analysis based in differential genes in tumor samples versus NAT of LUAD patients from TCGA LUAD cohort (n = 585), which were downloaded from the University of California Santa Cruz browser (https://xenabrowser.net/).

e. Detailed network map of the disturbed metabolic pathways showing the alterations of metabolites, mRNA and proteins levels in invasive LUAD tumors versus NAT. Color corresponds to the $\log_2$ fold changes between tumor and NAT.

Supplementary Figure 5

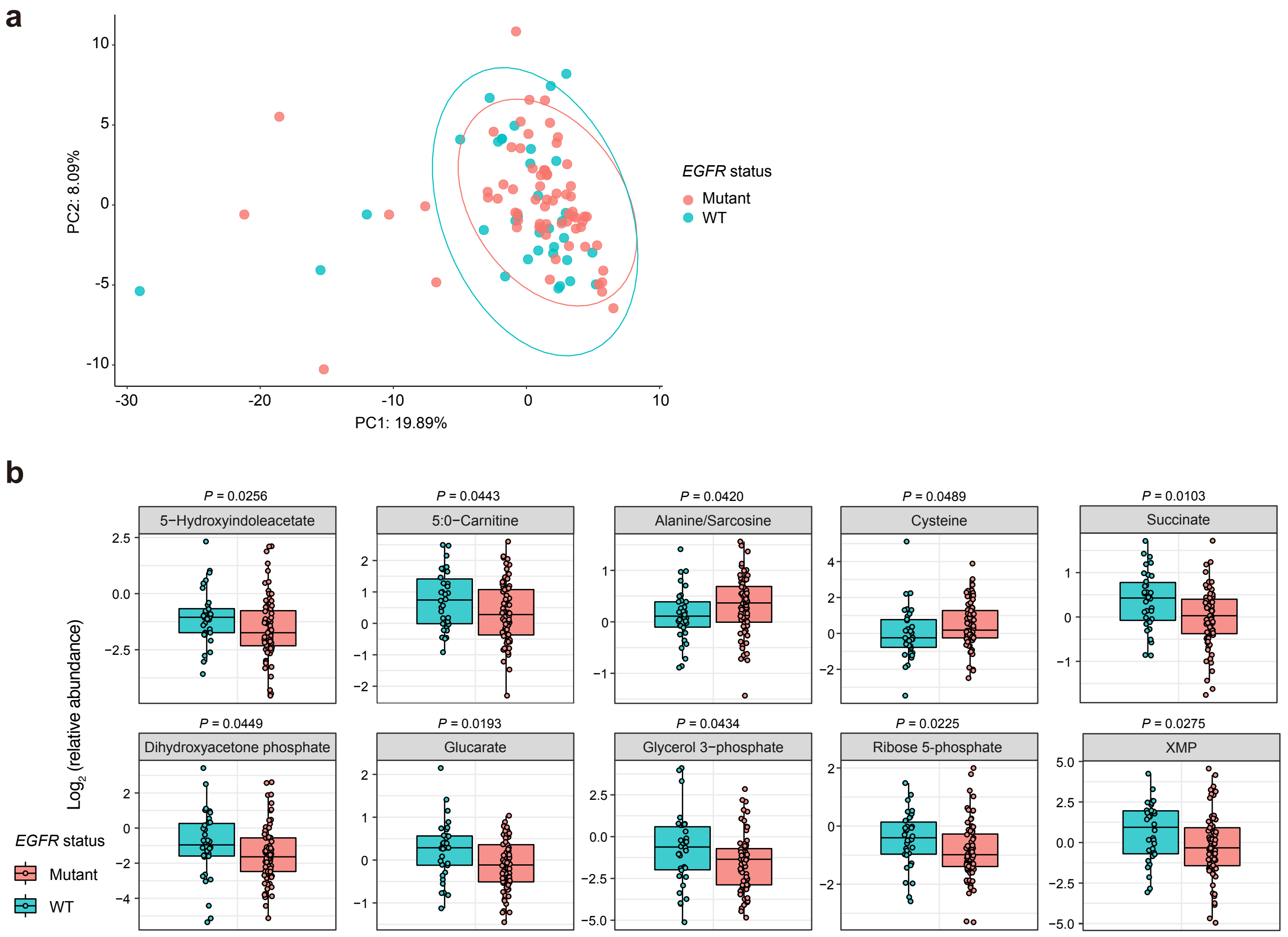

Supplementary Figure 5. Metabolomics characteristics of IAC patients with *EGFR* mutation

a. Principle component analysis (PCA) of metabolomics data in IAC patients with or without EGFR mutation.

b. Differentially altered metabolites in *EGFR*-mutant (n = 68) versus *EGFR*-WT (n = 34) tumors.

Two-sided Wilcoxon rank-sum tests with $P < 0.05$. The center line represents the median, and the box bound represents the inter-quartile range. Bars extend to 1.5-fold the inter-quartile range.

Supplementary Figure 6

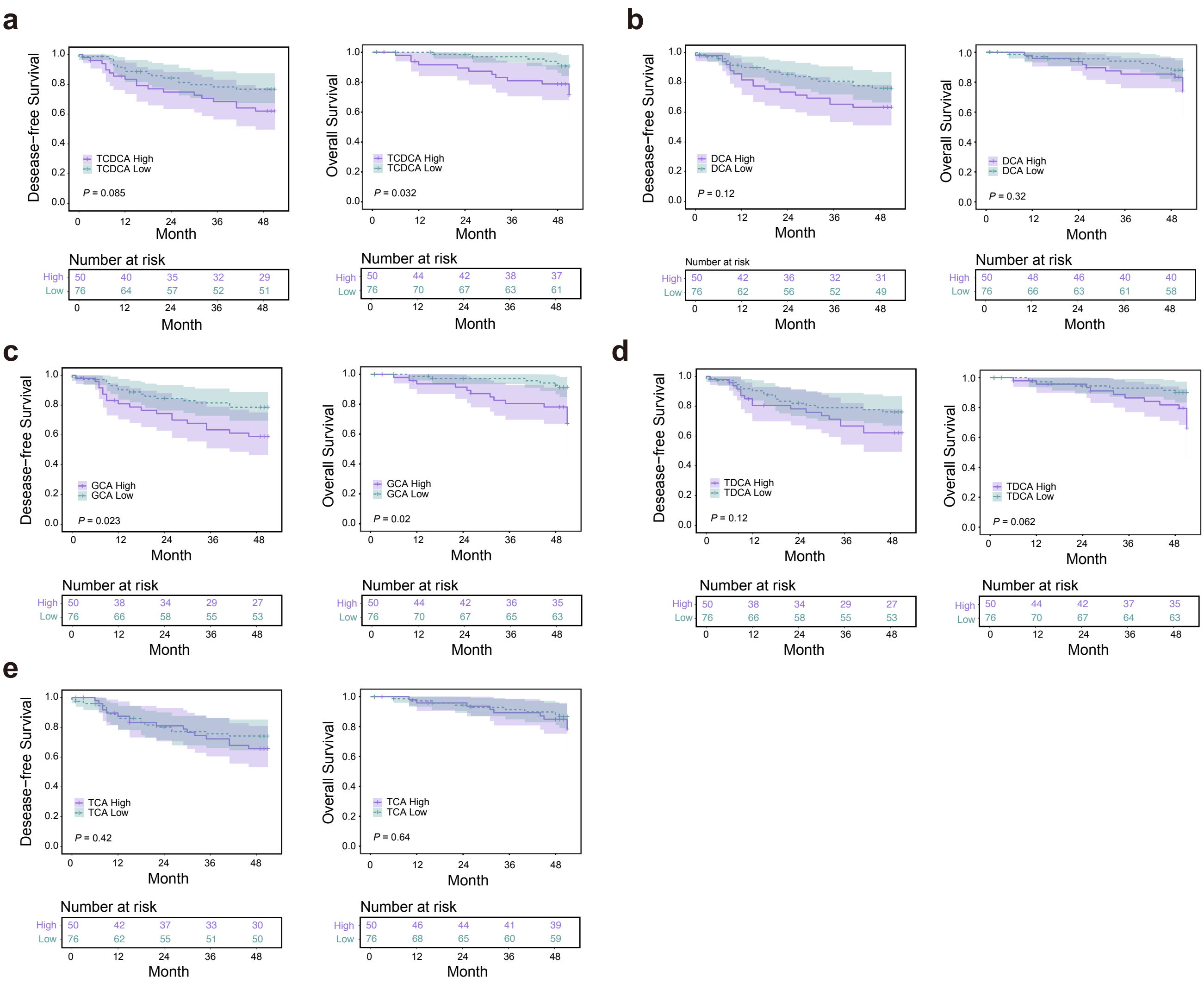

Supplementary Figure 6. The asscociation of bile acids with clinical outcomes.
Kaplan-Meier curves predicting the disease-free survival and overall survival of IAC patients stratified by the level of each bile acid with log rank P value. The patients were divided into high and low groups by 0.4 quantile of the bile acids levels in IAC patients.

Supplementary Figure 7

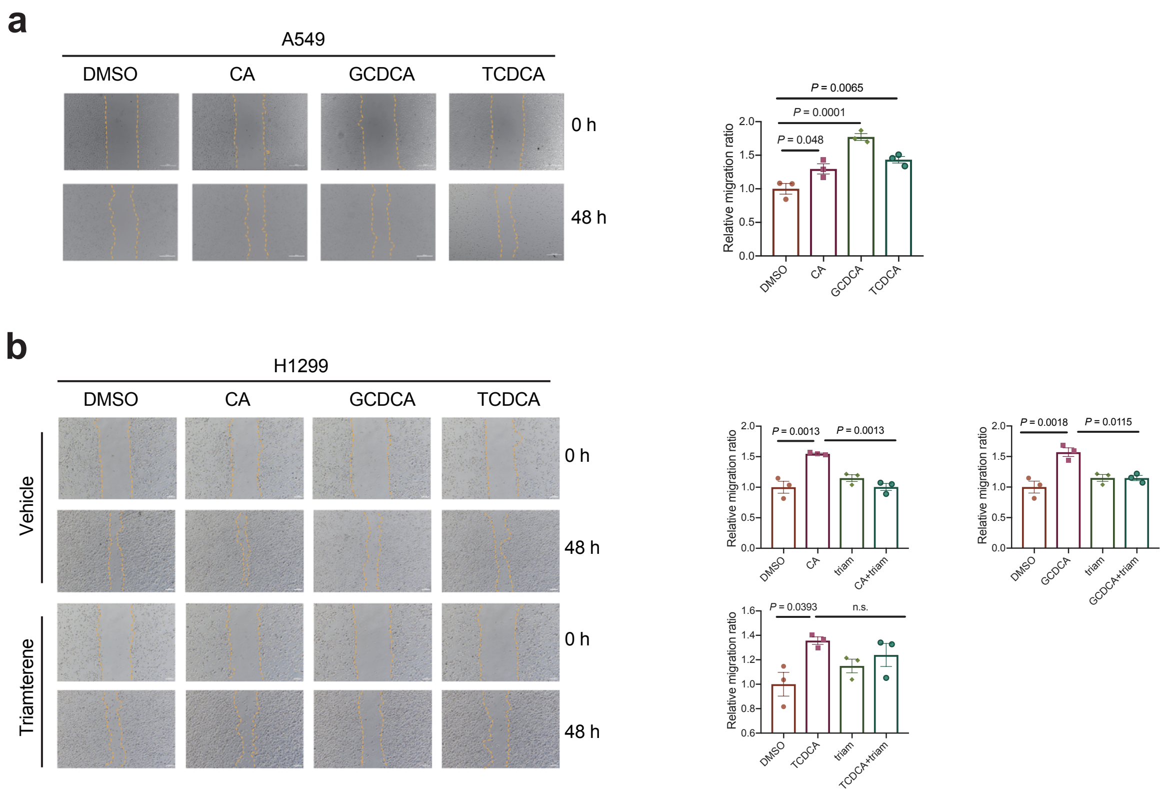

Supplementary Figure 7. Exogenous bile acid promoted cell migration.

a. Wound healing assay was performed to in A549 cells treated with CA (100 μM), TCDCA (100 μM) and GCDCA (100 μM) for 48 h. Representative images (left, 50 μm) for three biological repeats and quantification (right, n = 3) of wound closure are shown.

b. Wound healing assay was performed in H1299 cells treated with CA (100 μM), TCDCA (100 μM) and GCDCA (100 μM), with or without Triamterene (10 μM) for 48 h. Representative images (left, 50 μm) for three biological repeats and quantification (right, n = 3) of wound closure are shown.

Data represent the mean ± s.e.m., and One-way ANOVA followed by Tukey's multiple comparison test was used.

Supplementary Figure 8

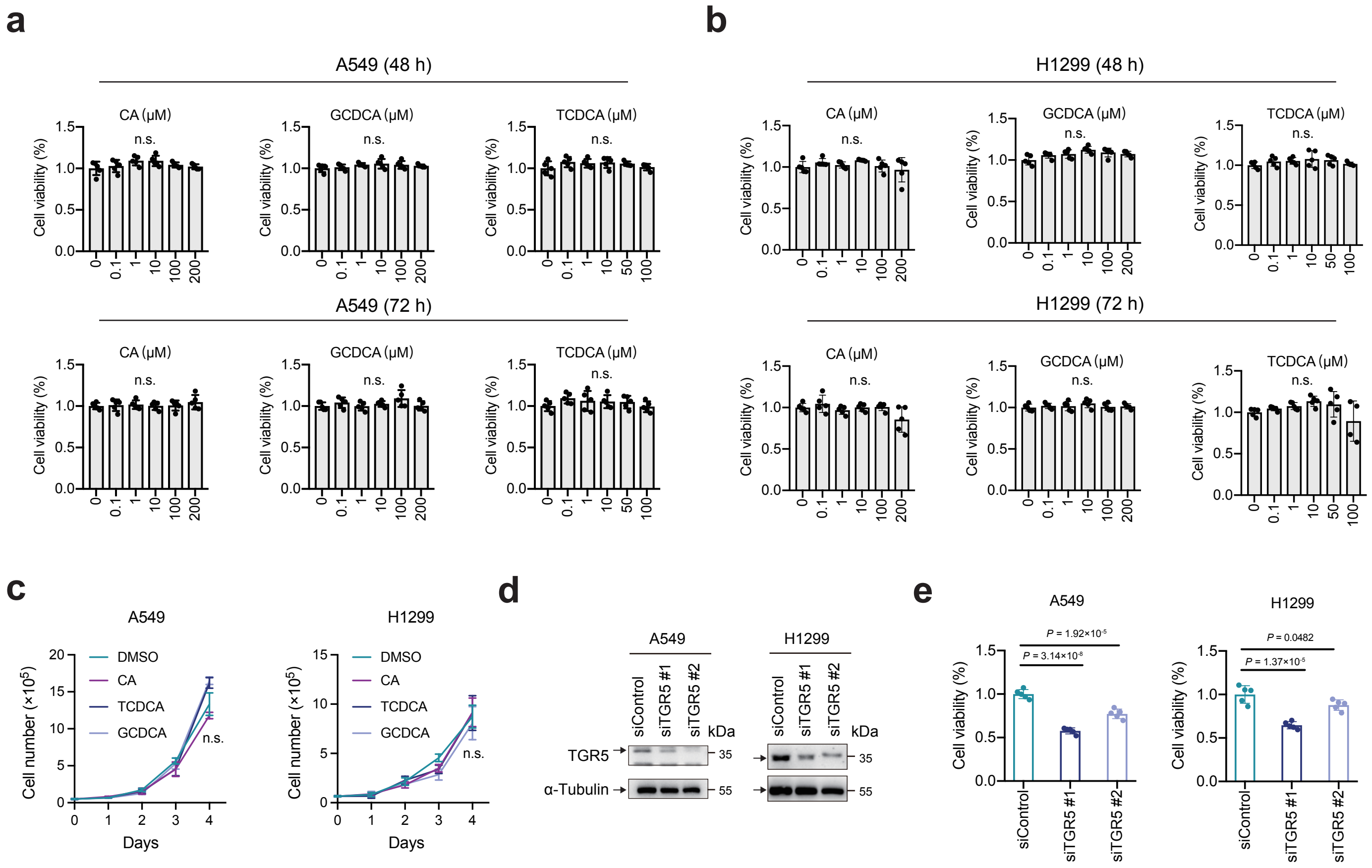

Supplementary Figure 8. The effect of manipulating bile acid metabolism on cell growth.

a, b. Cell viability of A549 and H1299 cells treated with CA, GCDCA, and TCDCA in the indicated concentrations at 48 h and 72 h (n = 5).One-way ANOVA followed by Tukey's multiple comparison test was used.

c. Cell proliferation of A549 and H1299 cells treated with CA (100 μM), GCDCA (100 μM), and TCDCA (100 μM) at the indicated time (n = 3). Data represent mean ± s.e.m. of three biologically independent experiments, and One-way ANOVA followed by Tukey's multiple comparison test was used.

d. Protein expression in A549 and H1299 cells treated with indicated siRNAs were analysed by western blot. The experiment was performed three times independently with similar results.

e. Cell viability of A549 and H1299 cells treated with indicated siRNAs (n = 5).

Data represent mean ± s.e.m., One-way ANOVA followed by Tukey's multiple comparison test was used.

Supplementary Figure 9

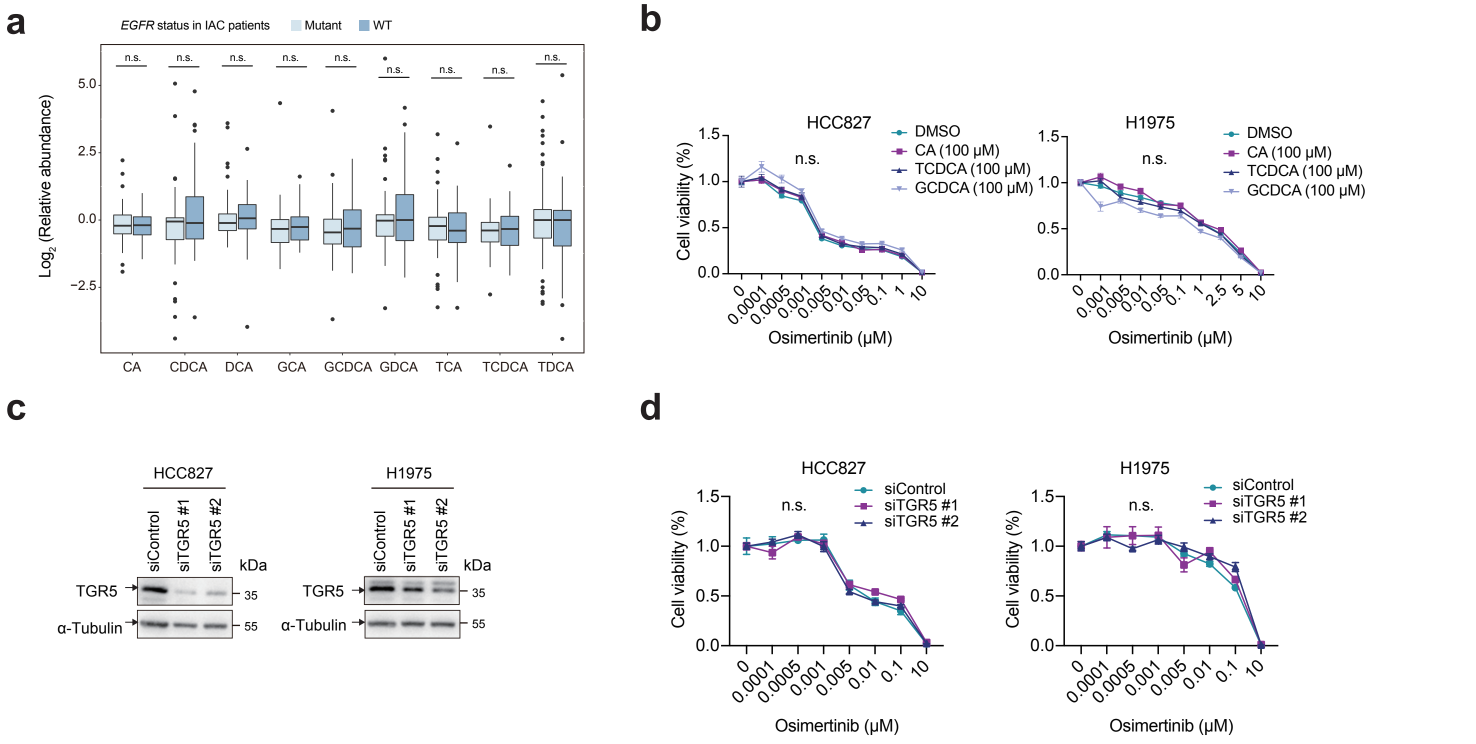

Supplementary Figure 9. Bile acid metabolism may not associate with drug response of *EGFR* TKIs.
a. Bile acid levels in IAC patients with *EGFR*-mutation (n = 66) and *EGFR*-WT (n = 34). Two-sided Wilcoxon rank-sum test was used. The center line represents the median, and the box bound represents the inter-quartile range. Bars extends to 1.5-fold the inter-quartile range.
b. Dose response curves of HCC827 and H1975 cells incubated in increasing concentrations of Osinertinib and treated with different bile acids for 72 h (n = 6). IC50 values were compared by One-way ANOVA followed by Dunnett's multiple comparison test. **c**. Protein expression in HCC827 and H1975 cells treated with indicated siRNAs were analysed by western blot. The experiment was performed three times independently with similar results.
d. Dose response curves of HCC827 and H1975 cells incubated in increasing concentrations of Osinertinib and treated with indicated siRNAs (n = 6). IC50 values were compared by One-way ANOVA followed by Dunnett's multiple comparison test. For **b** and **d**, data represent mean ± s.e.m. of one out of two independent experiments.

Supplementary Table 1

	siRNA targeting sequence
siTGR5 #1	5’-CCUGUACCUCGAAGUCUAUTT-3’
siTGR5 #2	5’-UCGUCUACUUGGCUCCCAATT-3’
