# Supplementary material for: Evolutionary metabolic landscape from preneoplasia to invasive lung adenocarcinoma
Source: Nat Commun. 2021 Nov 10;12:6479. doi: 10.1038/s41467-021-26685-y (PMC8580984; doi:10.1038/s41467-021-26685-y)
Supplement: Supplementary file 2 — Description of Additional Supplementary Files [file 41467_2021_26685_MOESM2_ESM.pdf]

Description of Additional Supplementary Files

Supplementary Data 1: Clinical information of cohort 1 and cohort 2.

Supplementary Data 2: Raw metabolomics mass spectrometry data of clinical cohort 1 and cohort 2.

Supplementary Data 3: Normalized metabolomics data of clinical cohort 1 and cohort 2.

Biomedicine Hall, RM C-312, Tsinghua University, Beijing 100084, China
Office: +86-10-6279 9839; Cell: +86 136 9368 7577. Email: zeping_hu@tsinghua.edu.cn
