# Supplementary material for: Evolutionary metabolic landscape from preneoplasia to invasive lung adenocarcinoma
Source: Nat Commun. 2021 Nov 10;12:6479. doi: 10.1038/s41467-021-26685-y (PMC8580984; doi:10.1038/s41467-021-26685-y)
Supplement: Supplementary file 6 — Reporting summary [file 41467_2021_26685_MOESM6_ESM.pdf]

nature research

Corresponding author(s): Peng Zhang, Zeping Hu

Last updated by author(s): Sep 3, 2021

Reporting Summary

Nature Research wishes to improve the reproducibility of the work that we publish. This form provides structure for consistency and transparency in reporting. For further information on Nature Research policies, see our Editorial Policies and the Editorial Policy Checklist.

Statistics

For all statistical analyses, confirm that the following items are present in the figure legend, table legend, main text, or Methods section.

n/a	Confirmed	
☐	☒	The exact sample size (*n*) for each experimental group/condition, given as a discrete number and unit of measurement
☐	☒	A statement on whether measurements were taken from distinct samples or whether the same sample was measured repeatedly
☐	☒	The statistical test(s) used AND whether they are one- or two-sided<br>*Only common tests should be described solely by name; describe more complex techniques in the Methods section.*
☒	☐	A description of all covariates tested
☐	☒	A description of any assumptions or corrections, such as tests of normality and adjustment for multiple comparisons
☐	☒	A full description of the statistical parameters including central tendency (e.g. means) or other basic estimates (e.g. regression coefficient) AND variation (e.g. standard deviation) or associated estimates of uncertainty (e.g. confidence intervals)
☐	☒	For null hypothesis testing, the test statistic (e.g. *F*, *t*, *r*) with confidence intervals, effect sizes, degrees of freedom and *P* value noted<br>*Give P values as exact values whenever suitable.*
☒	☐	For Bayesian analysis, information on the choice of priors and Markov chain Monte Carlo settings
☐	☒	For hierarchical and complex designs, identification of the appropriate level for tests and full reporting of outcomes
☐	☒	Estimates of effect sizes (e.g. Cohen's *d*, Pearson's *r*), indicating how they were calculated

Our web collection on statistics for biologists contains articles on many of the points above.

Software and code

Policy information about availability of computer code

Data collection	Targeted metabolomics data were collected with the Xevo TQ-XS mass spectrometer (Waters). Detection of bile acids was performed with a triple quadrupole mass spectrometer (Qtrap 6500+, SCIEX).
Data analysis	Skyline 4.1 software (MacCoss Lab)<br>SIMCA-P software version 14.1<br>MultiQuant software version 3.0 (SCIEX)<br>Mfuzz version 2.46.0<br>ConsensusClusterPlus version 1.50.0<br>SPSS software version 27.0<br>survival version 3.2-11<br>clusterProfiler version 3.14.3<br>edgeR version 3.28.1<br>R survminer version 0.4.9<br>Bioluminescence imaging analysis: IVIS-Image software version 4.3.1<br>GraphPad Prism version 8.2.1<br>Analyst Instrument Control software v.1.6.3 (SCIEX)

For manuscripts utilizing custom algorithms or software that are central to the research but not yet described in published literature, software must be made available to editors and reviewers. We strongly encourage code deposition in a community repository (e.g. GitHub). See the Nature Research guidelines for submitting code & software for further information.

Data

Policy information about availability of data

All manuscripts must include a data availability statement. This statement should provide the following information, where applicable:

- Accession codes, unique identifiers, or web links for publicly available datasets
- A list of figures that have associated raw data
- A description of any restrictions on data availability

Clinical information of patients with different stages are included in Supplementary Data1. Raw metabolomics mass spectrometry data are included in Supplementary Data 2. Normalized metabolomics data are included in Supplementary Data 3. The GDC TCGA LUAD dataset can be downloaded from https://xenabrowser.net/datapages/. The previously published RNA-seq data and proteomics data can be obtained from DOI: 10.1016/j.cell.2020.05.043. Raw data for plotting figures are provided in Source Data file. Source Data are provided with this paper.

Field-specific reporting

Please select the one below that is the best fit for your research. If you are not sure, read the appropriate sections before making your selection.

- [x] Life sciences
- [ ] Behavioural & social sciences
- [ ] Ecological, evolutionary & environmental sciences

For a reference copy of the document with all sections, see nature.com/documents/nr-reporting-summary-flat.pdf

Life sciences study design

All studies must disclose on these points even when the disclosure is negative.

Sample size	No statistical methods were used to pre-determine sample size. Because surgical resection is not the standard of care, the specimens from lung adenocarcinoma precursors were scarcity. The sample size of patients are adequate compared to published papers in the field (PMID: 33976164, PMID: 31784532) and sufficient for statistical analysis.
Data exclusions	No data were excluded from the analysis.
Replication	Biological replications are as indicated in figure legends and methods. All attempts at replication were successful.
Randomization	For targeted metabolomics studies, patient samples including tissue and plasma were randomized into different sampling batches. For all animal studies, mice were randomized into different research groups.
Blinding	Each sample was labeled with an numeric ID whose annotation was kept blinded during data collection and analyses.

Reporting for specific materials, systems and methods

We require information from authors about some types of materials, experimental systems and methods used in many studies. Here, indicate whether each material, system or method listed is relevant to your study. If you are not sure if a list item applies to your research, read the appropriate section before selecting a response.

Materials & experimental systems

n/a	Involved in the study	
[ ]	[x]	Antibodies
[ ]	[x]	Eukaryotic cell lines
[x]	[ ]	Palaeontology and archaeology
[ ]	[x]	Animals and other organisms
[ ]	[x]	Human research participants
[x]	[ ]	Clinical data
[x]	[ ]	Dual use research of concern

Methods

n/a	Involved in the study	
[x]	[ ]	ChIP-seq
[x]	[ ]	Flow cytometry
[x]	[ ]	MRI-based neuroimaging

Antibodies

Antibodies used	The antibody was purchased from the indicated source: TGR5 (Abcam, ab72608), vimentin (Proteintech, 10366-1-AP),α-Tubulin (ABclonal, catalog No: AC012)
Validation	The antibody used in this work were purchased from company, and validated by the manufacturers and by extensive use in published work.

Eukaryotic cell lines

Policy information about cell lines

Cell line source(s)	Human NSCLC cell lines H1299, A549, HCC827 and H1975 were purchased from ATCC.
Authentication	Cell line identity was confirmed by short-tandem repeat analysis.
Mycoplasma contamination	The cell line was tested for mycoplasma contamination prior to commencement of experiments and was negative.
Commonly misidentified lines (See ICLAC register)	No commonly misidentified cell lines were used in this study.

Animals and other organisms

Policy information about studies involving animals; ARRIVE guidelines recommended for reporting animal research

Laboratory animals	nu/nu athymic mice, female, 8 weeks. The mice were maintained on a 12-hr/12-hr light/dark cycle throughout the experiment. Temperature was set to 20-24°C and humidity at 40%-70%.
Wild animals	This study did not involve wild animals.
Field-collected samples	This study did not involve field-collected samples.
Ethics oversight	All experimental procedures were approved by the Animal Care and Use Committee of Tsinghua University.

Note that full information on the approval of the study protocol must also be provided in the manuscript.

Human research participants

Policy information about studies involving human research participants

Population characteristics	We enrolled two independent cohorts in our study. Tissue samples were obtained from a total of 181 patients (66 male, 115 female) in cohort 1, including 12 atypical adenomatous hyperplasia (AAH), 22 adenocarcinoma in situ (AIS), 19 minimally invasive adenocarcinoma (MIA) and 128 invasive adenocarcinoma (IAC) patients. Plasma samples were obtained from a total of 92 patients (18 male, 74 female) in cohort 2, including 10 patients with benign diseases, 32 AIS, 22 MIA, and 28 IAC patients. More information including age were provided in Methods and Supplementary Data 1.
Recruitment	The patient tissue specimens for this study were collected from Shanghai Pulmonary Hospital during December 2015 to April 2016. The patient plasma samples for this study were collected from Shanghai Pulmonary Hospital during February 2021 to March 2021. Patients receiving any anti-cancer treatments before surgery were excluded. There was no selection bias.
Ethics oversight	The study was approved by the Research Ethics Committee of Shanghai Pulmonary Hospital (Institutional Review Board: K20-317), and written informed consent was obtained from each patient.

Note that full information on the approval of the study protocol must also be provided in the manuscript.
